# Dynamic Time-Locking Mechanism in the Cortical Representation of Spoken Words

**DOI:** 10.1523/ENEURO.0475-19.2020

**Published:** 2020-07-22

**Authors:** A. Nora, A. Faisal, J. Seol, H. Renvall, E. Formisano, R. Salmelin

**Affiliations:** 1 Department of Neuroscience and Biomedical Engineering, and Aalto NeuroImaging, Aalto University, Espoo FI-00076, Finland; 2 Department of Cognitive Neuroscience, Faculty of Psychology and Neuroscience, Maastricht University, Maastricht 6200 MD, The Netherlands; 3 Maastricht Center for Systems Biology (MaCSBio), Maastricht University, Maastricht 6200 MD, The Netherlands

**Keywords:** auditory system, magnetoencephalography, neural decoding, speech processing

## Abstract

Human speech has a unique capacity to carry and communicate rich meanings. However, it is not known how the highly dynamic and variable perceptual signal is mapped to existing linguistic and semantic representations. In this novel approach, we used the natural acoustic variability of sounds and mapped them to magnetoencephalography (MEG) data using physiologically-inspired machine-learning models. We aimed at determining how well the models, differing in their representation of temporal information, serve to decode and reconstruct spoken words from MEG recordings in 16 healthy volunteers. We discovered that dynamic time-locking of the cortical activation to the unfolding speech input is crucial for the encoding of the acoustic-phonetic features of speech. In contrast, time-locking was not highlighted in cortical processing of non-speech environmental sounds that conveyed the same meanings as the spoken words, including human-made sounds with temporal modulation content similar to speech. The amplitude envelope of the spoken words was particularly well reconstructed based on cortical evoked responses. Our results indicate that speech is encoded cortically with especially high temporal fidelity. This speech tracking by evoked responses may partly reflect the same underlying neural mechanism as the frequently reported entrainment of the cortical oscillations to the amplitude envelope of speech. Furthermore, the phoneme content was reflected in cortical evoked responses simultaneously with the spectrotemporal features, pointing to an instantaneous transformation of the unfolding acoustic features into linguistic representations during speech processing.

## Significance Statement

It has remained unclear how speech is processed differently from other sounds with comparable meanings and spectrotemporal characteristics. In this study, computational modeling of cortical responses to spoken words highlights the relevance of temporal tracking of spectrotemporal features especially for speech. This mechanism is likely pivotal for transforming the acoustic-phonetic features into linguistic representations.

## Introduction

Humans effortlessly recognize and react to natural sounds but are especially tuned to speech. Numerous studies have attempted to localize speech-specific processing stages in the brain (Price et al., 2005; Zatorre and Gandour, 2008; Schirmer et al., 2012), but while subtle differences in the time sequence of activation for processing speech versus other meaningful sounds have been described (Salmelin, 2007; Renvall et al., 2012), it remains unclear whether and how they relate to possible unique computations in speech processing. A major challenge has been to describe how the brain matches highly variable acoustic signal to linguistic representations, when there is no one-to-one correspondence between the two. Superior temporal cortices show sensitivity to spectrotemporal features of speech that correspond to different phonemes (Chang et al., 2010; Mesgarani et al., 2014), and to temporal structure of speech sounds but not other sounds with similar acoustic content (Overath et al., 2015). Thus, detailed tracking of temporal modulations that distinguish between phonemes may be crucial for speech processing (Poeppel, 2003; Zatorre and Gandour, 2008).

Recently, temporal locking (“entrainment”) of cortical oscillations to the amplitude envelope of speech has emerged as a potential mechanism for mapping between multiscale spectrotemporal features and linguistic structures (Ding and Simon, 2014; Ding et al., 2016; Obleser and Kayser, 2019). Low-frequency cortical oscillatory activation to speech appears to be best modeled as a combination of spectrotemporal features and phonetic-feature labels, highlighting tracking of spectrotemporal detail within speech (Di Liberto et al., 2015). However, whether temporal tracking, by continuous oscillatory activation or evoked responses as used here, is particularly relevant for processing of speech, and less crucial for other types of sounds, remains an open question. Subcortical and cortical neurons, starting from the cochlea, show phase-locked encoding of both environmental and speech sounds (Worden and Marsh, 1968; Coffey et al., 2016), and oscillatory entrainment is observed also for other sounds than speech, for example music (Doelling and Poeppel, 2015). In contrast, recent studies have suggested that spectrotemporal modulations corresponding to different phonetic features might not be best represented in a linear, time-locked manner (Chi et al., 2005; Pasley et al., 2012). Indeed, accurate reconstruction of speech can be achieved from functional magnetic resonance imaging (fMRI) recordings by modeling the encoding of the speech signal as frequency-specific spectrotemporal modulation filters, despite the loss of temporal information due to the sluggish hemodynamics and poor temporal sampling of the blood oxygen level-dependent (BOLD) response (Santoro et al., 2014, 2017). This suggests spatially distributed representations of temporal information, similarly to frequency content, and questions the crucial role of temporal tracking of the speech signal.

The question of whether there is something special in the cortical processing of speech has often been approached through careful acoustic matching of linguistic and nonlinguistic stimuli and comparison of their activation patterns in the brain (Scott et al., 2000; Peelle et al., 2013; Overath et al., 2015). Here, we take a different approach: Our stimuli are natural spoken words and environmental sounds and, instead of delimiting their acoustic properties, we make full use of their large natural variability. We evaluate the hypothesis that temporal tracking of the spectrotemporal content is particularly important in cortical encoding of spoken words by modeling and decoding the sounds, using multiple physiologically-inspired representations that differ in temporal detail. Cortical activation is measured with magnetoencephalography (MEG), which detects cortex-wide neuronal signaling on a millisecond scale. Decoding of the time-varying features of the sounds is achieved using a convolution model (Bialek et al., 1991; Mesgarani et al., 2009; Pasley et al., 2012; Faisal et al., 2015), with a new formulation that efficiently handles the high spatiotemporal dimensionality of MEG data. The convolution model assumes that the activation of neuronal populations follows closely in time the time sequence of stimulus features. It is compared with a regression model where no such time-locking is assumed (Mitchell et al., 2008; Sudre et al., 2012; Santoro et al., 2014). The ability of each model to decode and reconstruct the sounds, using variations in the MEG signal, reveals whether the underlying assumptions of the model are an accurate description of how the human brain encodes the stimulus features. Importantly, this approach does not require a direct comparison of cortical responses to speech versus other sounds. Earlier studies that have combined machine learning-based neural decoding models (Mitchell et al., 2008) with models of cortical processing of sounds (Chi et al., 2005) have demonstrated tonotopy as well as selectivity for specific spectrotemporal modulations in the auditory cortical areas (Santoro et al., 2014), and allowed successful reconstruction of spoken word acoustics based on fMRI and intracranial recordings (Pasley et al., 2012; Santoro et al., 2017; Akbari et al., 2019). Phoneme categories or articulatory features have been shown to be reflected in EEG, fMRI, and intracranial recordings and have been decoded with quite good accuracy (Formisano et al., 2008; Chang et al., 2010; Mesgarani et al., 2014; Di Liberto et al., 2015), while semantic classification of spoken words has so far been reported only for small sets of stimuli (Simanova et al., 2010; Chan et al., 2011; Correia et al., 2015) or based on dissimilarity to preceding context (Broderick et al., 2018). The current work is the first to address acoustic, phonological, and semantic levels of analysis in the same study and to systematically evaluate the same time-varying and non-time-varying decoding models for spoken words and environmental sounds that refer to corresponding meanings (e.g., the uttered word “cat,” and the sound of a cat “meow”). This study thus offers essential new insights into cortical mechanisms that are potentially particularly relevant for the encoding of speech, as compared with other sounds.

## Materials and Methods

### Participants

The participants were 16 (eight male, eight female) right-handed Finnish-speaking volunteers aged 19–35 years (mean 24 years). Exclusion criteria were hearing disorders and developmental or acquired neurologic or language disorders. Participants signed a written consent form before the measurement, in agreement with the prior approval of the University’s research ethics committee. The originally planned sample size was 20 participants, but data from only 16 was collected due to practical reasons. While the sample size could be considered relatively small according to current neuroimaging standards, the observed effects were robust in all individuals, with effect size ranging from moderate to large.

### Stimuli and experimental design

The speech stimuli were 44 words from various semantic categories (Extended Data Fig. 1-1 and Table 1-1). To increase acoustic variability, each word within a category was spoken by a different speaker (eight speakers in total; four females, four males; two children/adolescents). Also, each speaker spoke one word in each semantic category, to enable eliminating the influence of speaker-specific acoustic features from category-level decoding. The speaker set was rotated across participants. Stimulus duration was on average 810 ms (SD 180 ms) for the spoken words and 920 ms (SD 230 ms) for the environmental sounds. The spoken words were composed of two to five syllables, with a few compound words included. The uniqueness point (i.e., estimated time of lexical selection) was on average 500 ms (range 300–890 ms, from first to fourth syllable). Because of the transparent nature of Finnish, the uniqueness point of a spoken word (point of divergence from all other words with a different word stem) corresponds to its orthographic uniqueness point, calculated here based on the same 1.5 billion-token Finnish Internet-derived text corpus that was used to create the semantic features (Kanerva and Ginter, 2014).

**Figure 1.**
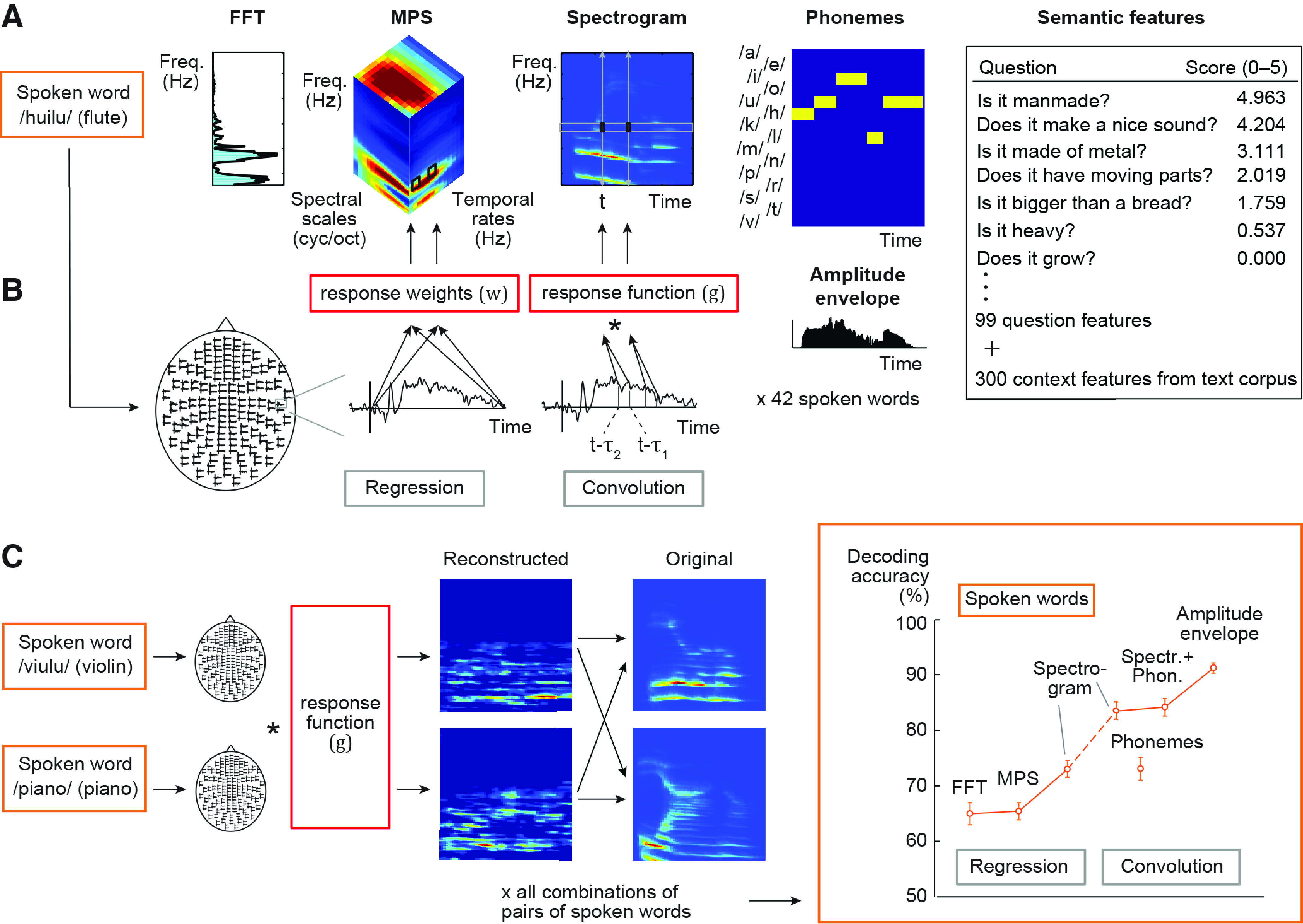
Different decoding models and their prediction accuracy. ***A***, Stimulus features for a spoken word. The FFT model represents the frequency spectrum of the sound extracted in 128 frequency bands with logarithmically spaced center frequencies. The MPS model represents energy in four spectral scales (wide to narrow) and four temporal modulation rates (slow to fast), averaged over time. The sound spectrogram quantifies the time-evolving frequency content of the sound extracted in 128 frequency bands and 10-ms time windows by using short-term FFT. The amplitude envelope carries the temporal changes without frequency information (shown below the phoneme model). The phoneme sequence is the phoneme annotation of the word for each 10-ms time window. Semantic features were represented by scores of 99 questions (a few example questions shown) and a 300-dimensional vector trained with the co-occurrences of context words (words occurring near the stimulus words) in a large text corpus. ***B***, Model estimation for the regression model (left) and the convolution model (right). A mapping between the cortical MEG responses (here illustrated on one sensor), and each stimulus feature was learned with kernel regression or kernel convolution model. As illustrated here, the regression model predicts, for example, power at each frequency-rate-scale point of the MPS by multiplying cortical responses at all (or selected) time points with unknown weights (w). The convolution model predicts the amplitude at each time-frequency point of the spectrogram by convolving the time-sequence of cortical responses with an unknown spatiotemporal response function (g). Specifically, values at each frequency band of a new spoken word are predicted at each time point *t* (moving from 0 to end of the sound) based on MEG responses in the time range from (*t* – *τ*_2_) to (*t* – *τ*_1_), here illustrated for the lag window –*τ*_2_ = 100 to –*τ*_1_ = 180 ms at time points *t*. ***C***, Model testing aimed to tell apart two left-out sounds by reconstructing the sound features (here, spectrogram) and correlating them with the original features. The procedure was repeated for all possible pairings of sounds. Predictive accuracy for the spoken words across all test sound pairs and 16 participants (mean ± SEM) is shown on the right. The regression model was used for the decoding of non-time-varying features (FFT frequency bins/MPS rate-scale-frequency points/semantic question scores and corpus statistics), and the convolution model was used for decoding of time-varying features (spectrogram time-frequency points/phonemes at each time point/amplitude envelope); for a control analysis, the spectrogram was also decoded with the regression model. Predictive accuracy improved markedly when spoken words were modeled with the convolution spectrogram model, formalizing the concept that the neuronal population response follows closely in time the unfolding time-sequence of the sound acoustics. Even better performance was obtained when the spoken words were modeled using both the spectrogram and the phoneme sequence descriptions, and the best performance was obtained using the sounds’ amplitude envelope.

10.1523/ENEURO.0475-19.2020.f1-1 Extended Data Figure 1-1 Examples of spoken words (left) and environmental sounds (right), one from each semantic category. For each sound, the signal waveform is depicted on top, with the three models of the sound below: frequency spectrum (FFT), spectrogram, and MPS (temporal rates and spectral scales shown here separately). Note the considerable variability in the spectral content and temporal evolution of the different sounds, especially for the environmental sounds. Download Figure 1-1, EPS file.

10.1523/ENEURO.0475-19.2020.t1-1 Extended Data Table 1-1 Table containing a complete list of stimulus items. Download Table 1-1, DOC file.

The environmental sounds were high-quality sounds chosen from Internet sound libraries and, using the Adobe Audition program, modified to readily identifiable mono sounds of ∼1-s duration, with sampling frequency 44.1 kHz and bit rate of 16 bits. With careful selection of sounds from several categories, we sought to include as much acoustic variability as possible to extensively span the low-level acoustic feature space. The experimental stimuli were selected from these sounds based on a behavioral test with 12 participants (who did not participate in the MEG study): participants wrote down the name of each sound immediately after recognizing it. Environmental sounds with at least 80% consistent naming and response time <3 s were chosen as stimuli. The final 44 environmental sound stimuli belong to six categories: animals, human sounds, tools, vehicles, musical instruments and others (six to eight items per category, see Extended Data Fig. 1-1 and Table 1-1; the spoken word stimuli were the noun labels of the final selection of environmental sounds). The others category included items that did not belong to any of the other above-mentioned categories. The same environmental sounds were used as stimuli across all participants.

All sounds were filtered with an 8-kHz linear low-pass fast Fourier transform (FFT) filter (Blackman–Harris) and re-sampled at 16 kHz. Mean amplitudes of the stimuli were normalized such that the root-mean-square power of each stimulus was the same. Before the MEG measurement, the individual hearing threshold was determined for each participant, and the stimuli were delivered through plastic tubes and earpieces at 75 dB (sensation level).

To reach a high enough MEG signal-to-noise ratio (SNR) per stimulus item for the machine learning approach, each stimulus was presented 20 times in a pseudorandom manner, such that two words spoken by the same speaker, or a spoken word and an environmental sound referring to the same meaning were not presented in a row. Event-related fields were calculated as an average of these 20 repetitions. To ensure concentration, participants performed a one-back task: They were instructed to listen carefully to each sound, think about the concept it refers to, and respond with a finger lift when two sounds with the same meaning were presented one after another (4% of trials). Response hand was alternated between participant pairs. In the task trials, another exemplar of a word or an environmental sound was presented as target stimulus (same word spoken by a different speaker or same environmental sound with a different acoustic form, e.g., a different kind of dog bark; the two stimulus types were not mixed, thus, e.g., the word cat was never followed by a cat sound).

Additional filler items, eight meaningful environmental sounds and nine spoken words, were included to make the sequence more variable. In addition, eight meaningless environmental sounds and eight pseudowords were presented 20 times each to increase participants’ attention. The meaningless environmental sounds were sounds from the abovementioned categories, which were further processed by reversing them in the time domain and, for some words, also scrambling them in 50- to 100-ms segments, to make them unidentifiable. The resulting sounds shared properties with the meaningful sounds from the same categories, but they were not identifiable in the behavioral pre-test. The pseudowords were minimal pairs to real Finnish words, following Finnish phonotactic rules. The task trials, filler sounds and meaningless sounds were excluded from the main analysis. However, we separately investigated pseudoword decoding with the different models to explore the possible contribution of lexical and semantic aspects in spoken word decoding.

### Acoustic, semantic, and phoneme features of the sounds

#### Acoustic features

We compared four sets of acoustic features that vary with respect to whether and how they incorporate information about the temporal modulations within the spoken words (Fig. 1*A*). The simplest set of features was the frequency spectrum, which is a non-time-varying representation of the stimulus power per frequency. FFT was calculated over the entire sound time window by using a filter bank of 128 frequency bands, with central frequencies of the bands ranging from 180 to 7246 Hz, uniformly distributed along a logarithmic frequency axis.

In addition to displaying an organization by frequency, the primary and secondary auditory cortices respond to different rates of temporal modulations at different spectral scales (Pasley et al., 2012; Santoro et al., 2014, 2017). These intricate multidimensional modulations within sounds are captured by our second set of features, the modulation power spectrum (MPS). The MPS [also called modulation transfer function (MTF), when describing neuronal filter properties; Elliott and Theunissen, 2009] represents energy in wide to narrow spectral scales and at slow to fast temporal modulation rates at different frequency bands (same as in FFT) for the entire duration of the sound (Chi et al., 2005). MPSs were calculated using the NSL toolbox (Chi et al., 2005) with modulation-selective filters spanning four spectral scales (0.5, 1, 2, and 4 cycles/octave) and four temporal modulation rates (1, 3, 9, and 27 Hz) within the sounds; these have been shown to capture the essential features of a broad range of natural sounds (Santoro et al., 2014). We chose a three-dimensional frequency-specific MPS where the time dimension of the MPS was averaged out, and upward-going and downward-going modulation rates were averaged together. This type of time-averaged modulation content has been shown to successfully decode environmental sounds from fMRI data (Santoro et al., 2014, 2017); computationally demanding time-varying MPS features are decodable from spatially limited intracranial recordings (Pasley et al., 2012).

For a time-evolving representation of the acoustic features, a spectrogram was generated from sounds using an auditory filter bank with 128 overlapping bandpass filter channels, with their central frequencies corresponding to the FFT frequency bands (following Pasley et al., 2012; http://nsl.isr.umd.edu/downloads.html). This filter bank mimics the representation of sound in the human cochlea (Chi et al., 2005). The time resolution and other parameters were optimized for retaining the characteristic temporal modulations of the sounds, while enabling extraction of the relevant spectral structure of the sounds. The sounds were divided into frames of 10 ms and integrated over 16-ms time windows.

Finally, we investigated a representation of the sounds that contains temporal modulation, but lacks fine spectral structure, by using the amplitude envelope of the sounds. The amplitude envelope was estimated through averaging the sound spectrogram across the 128 frequency bins in 10-ms windows, resulting in one feature vector.

The number of acoustic features were on average 87 in amplitude envelope (1 amplitude envelope × 87 time windows of 10 ms), 128 in FFT (128 frequency bands), 2048 in MPS (128 frequency bands × four spectral scales × four temporal modulation rates), and on average 11,136 in spectrogram (128 frequency bands × 87 time windows of 10 ms); for spectrogram and amplitude envelope, the length of the feature vectors varied with the sound length. To control for possible confounding effects of the varying lengths of feature vectors on the performance of the convolution model, the feature vectors for the two held-out test sounds (see below, Machine learning models and Performance evaluation, below) were always equalized to the length of the shorter one.

#### Phoneme sequence

Phoneme sequence of the words were obtained through their phonemic annotation, manually time-aligned to the stimulus wavefile using Praat software (Mesgarani et al., 2014; Di Liberto et al., 2015). This was a categorical representation, where each phoneme was set as 1 in those 10-ms time windows when it was present and otherwise as 0. There was no overlap, i.e., only one phoneme was marked “active” in each time window; it captures well the timing of phoneme onsets, but does not take coarticulation into account. Only phonemes with 10 or more instances in the stimulus set were included to reduce sparsity of the feature set, resulting in a set of 15 phonemes, each occurring 10–40 times. This representation had on average 1305 features.

#### Combined spectrogram and phoneme features

For investigating whether the spectrogram and phoneme decoding results were based on fully overlapping information, we combined these into a single feature representation of the spoken words, with on average 11,136 spectrogram features plus 1305 phoneme features; the number of features varied with sound length.

#### Semantic features

The semantic features were obtained by concatenating two sets of norms, one acquired through a questionnaire and the other using word co-occurrences in a large-scale text corpus. Question norms for the stimulus words were collected with a web-based survey. The questions in the survey were partly based on a previous study (Sudre et al., 2012) but modified to better represent the present selection of stimulus categories. Fifty-nine university students (32 female, 27 male, mean age 26 years; none of them participated in the present MEG study) answered 99 questions, presented in random order, about the semantic properties of each item on a scale from 0 to 5 (from definitely not to definitely yes); values for each question were averaged across the participants. Each item was thus described with a vector with 99 values.

For extracting the corpus statistics, the frequencies of co-occurrences of words in the immediate neighborhood (five words before and five words after) of each lemmatized stimulus word (compound markers added, some words changed from plural to singular) were calculated from a 1.5 billion-token Finnish Internet-derived text corpus (Kanerva and Ginter, 2014) using a continuous skip-gram Word2vec-algorithm using default parameters (Mikolov et al., 2013). The dimensionality of the trained vectors was 300, i.e., a vector with 300 values was used to describe each item.

#### Similarity measures

To investigate how the different acoustic models (especially the MPS and spectrogram models) differ in the dissimilarity among the speech versus non-speech sounds, we calculated the pairwise distances (1 minus sample correlation) for all spoken words and environmental sounds (Extended Data Fig. 3-1*B*,*D*). We chose correlation as our similarity measure, as correlation between the original sound and its reconstructed model was also used in the evaluation of the classification and reconstruction performance. Thus, this measure serves as a baseline measure of discriminability of the items with the different models, and for the spectrogram model it describes the cumulative dissimilarity of all time points at each frequency band of the spectrogram. To calculate the distances (1 minus correlation), the different frequency bands were concatenated.

#### Sound complexity measures

For estimating how the spectral distribution fluctuates across time in environmental sounds and spoken words we calculated, first, the variance of the spectral structure (spectral flatness measure SFM, an estimate of the number of distinct peaks in the frequency spectrum; Jayant and Noll, 1984) across each 10-ms segment within the spectrogram and, then, the spectral structure index (SSI; a measure of spectral variability defined as the SFM across time and suggested as a measure of sound complexity; Jayant and Noll, 1984). Larger values of SSI denote more variable spectral structure across time.

### MEG recording

Magnetic fields associated with neural current flow were recorded with a 306-channel whole-head neuromagnetometer (Elekta Oy). The sensor array consists of 102 triple sensor elements, each with one magnetometer and two planar gradiometers. Planar gradiometers are most sensitive to neural activity close to the sensors and give maximum signal directly above a current source; magnetometers also pick up signals from more far away sources. The MEG signals were acquired at 1000 Hz, with a hardware bandpass filter set between 0.03 and 330 Hz. Eye movements and blink artifacts were monitored by two diagonally placed electrodes measuring electro-oculogram signal (EOG). The position of the participant’s head within the MEG helmet was defined using five head position indicator coils. The locations of these coils, attached to the participant’s scalp, were determined with respect to three anatomic landmarks (nasion and two preauricular reference points) with a 3D digitizer, and with respect to the sensor array by briefly feeding current to the coils during the measurement. Head movements were monitored continuously (Uutela et al., 1999). The MEG measurement lasted ∼40 min, with breaks every 5 min.

### Anatomical MRI acquisition

Anatomical MRIs were obtained with a 3T MRI scanner (Magnetom Skyra, Siemens) for all 16 participants. The scan included a three-plane localizer and a T1-weighted anatomic image. To enable attribution of MEG activation patterns to cortical loci, the MEG data were co-registered in the same coordinate system with the individual MR images.

### MEG preprocessing and source modeling

Spatiotemporal signal space separation (tSSS; Taulu and Simola, 2006) and movement compensation algorithms (Uutela et al., 1999) were applied offline to the raw data using MaxFilter software (Elekta Neuromag), to remove the effects of external interference and to compensate for head movements during the measurement. To obtain an estimate of the artifact signals caused by blinks or saccades, the MEG signals were averaged with respect to transient maxima in the EOG signal, principal component analysis (PCA) was performed on this average, and the corresponding magnetic field component was removed from the raw data (Uusitalo and Ilmoniemi, 1997).

The MEG data analysis focused on planar gradiometer channels. Single trials were averaged from 300 ms before to 2000 ms after the stimulus onset, rejecting trials contaminated by any remaining artifacts (signal strength exceeding 3000 fT/cm). On average 19.7 ± 0.6 (mean ± SD) artifact-free epochs (trials) per participant were gathered per item (maximum = 20). The averaged MEG responses were baseline-corrected to the 300-ms interval immediately preceding the stimulus onset. For visualization purposes only, the averaged data were further low-pass filtered with the Elekta Neuromag Xplotter software, with a center frequency of 40 Hz and filter width of 10 Hz.

An estimate of the underlying cortical sources was obtained with minimum norm estimates (MNEs; Hämäläinen and Ilmoniemi, 1994) using MNE Suite software package (Gramfort et al., 2014). For MNE analysis, the cortical surface of each participant was reconstructed from their individual MR images with Freesurfer software (Dale et al., 1999; Fischl et al., 1999). Each hemisphere was covered with ∼5000 potential source locations. Currents oriented normal to the cortical surface were favored by weighting the transverse currents by a factor of 0.2, and depth-weighting was used to reduce the bias toward superficial sources (Lin et al., 2006). Noise-normalized MNEs [dynamical statistical parametric maps (dSPMs)] were calculated over the whole cortical area to estimate the SNR in each potential source location (Dale et al., 2000). Noise covariance matrix was estimated from the 300-ms prestimulus baseline periods across all trials. The source space (∼10,000 vertices) was divided into 229 parcels of approximately equal size (101 in the left and 118 in the right hemisphere), using the Destrieux Atlas as a starting point (Destrieux et al., 2010). Medial and orbitofrontal areas were excluded, as MEG does not reliably measure activation in these areas. Before applying the parcellation, the cortical surface of each participant was morphed onto Freesurfer’s average cortical surface template (fsaverage).

### Machine learning models

Each decoding model was trained and tested separately within each individual participant. Before model fitting, MEG sensor level responses were downsampled to 100 Hz (i.e., sampled at 10-ms resolution). All decoding models were trained and tested separately for the spoken words and for environmental sounds (training and testing within spoken words or environmental sounds, not mixed together). Decoding was performed both on sensor-level and source-level. In the sensor space, the analysis was restricted to 28 planar gradiometer pairs over the bilateral auditory cortices for the acoustic and phoneme decoding, whereas signals from all 204 planar gradiometers were used for semantic decoding. For decoding of time-varying features, i.e., the spectrogram frequencies, amplitude envelope and phoneme sequences, we used a convolutional model. For decoding of non-time-varying features, i.e., semantic features, frequency spectrum (FFT), and time-averaged MPS, we used a regression model.

Before performing machine learning analysis, the stimulus features and MEG signals were standardized across all stimuli, such that the mean value was set to zero and standard deviation to unity. For the acoustic features, the FFT and spectrogram were normalized within each frequency band, and the MPS within each rate, scale, and frequency band. Each semantic feature was similarly standardized across all stimuli. The phoneme sequences were categorical variables (with values 0/1), and thus not standardized. The MEG signal power per sensor or source location was normalized within each 10-ms time window. When applied to both stimulus features and corresponding MEG responses, this procedure ensures that the absolute power (e.g., per frequency band) does not affect model estimation. Instead, the models use variation in the MEG signal power to reconstruct variation in each stimulus feature, across stimuli. Data standardization is a normal practice in statistical analysis and ensures that all the different data features are weighted equally in the analysis (Friedman et al., 2001).

In the training phase, each model learns a mapping between a stimulus feature set and the MEG data based on all but two sounds (Fig. 1*A*,*B*). The model is then evaluated on the remaining data from the same participant, i.e., used to tell apart the two held-out words/environmental sounds based on their decoded features (leave-two-out cross-validation; Fig. 1*C*). The training and testing steps are repeated for all possible sound pairs.

We had two machine-learning models. Time-sensitive decoding of the acoustic features was approached with lagged linear regression, which convolves the time-varying signal with a multivariate response function, and non-time-sensitive decoding with regular regression. In the following, for simplicity, we will refer to these models as convolution and regression, respectively. Both the regression and convolution models decode acoustic features from multivariate spatiotemporal neural patterns, but the regression model does not model the potential temporal tracking of stimulus features in the neural responses.

The convolution model searches for a suitable mapping (stimulus response function) between the time-varying neural response and the time-varying stimulus features (Bialek et al., 1991; Mesgarani et al., 2009; Pasley et al., 2012; Faisal et al., 2015). Here, we use a scalable formulation of the model that allows decoding of time-varying properties of the sounds (*s*) by convolving the time-varying MEG responses (*r*) at brain location × with the spatiotemporal response function *g*. The model decodes stimulus features [${\hat{s}}_{f}$, where *f* is a given stimulus feature, here the amplitude at one spectral frequency band or the value (0/1) of one phoneme] at time *t*, based on the MEG activation (*r*) integrated from time (*t* – *τ*_2_) to (*t* – *τ*_1_); –*τ*_1_ > 0, –*τ*_2_ ≥ 0 (Fig. 1*B*, where –*τ*_1_ = 180 and –*τ*_2_ = 100). Thus, the convolution model decodes spectrogram at time *t* using neural responses at time (*t* –*τ*_2_) and at successive 10-ms time points until time (*t* – *τ*_1_). Thus, the lag values from stimulus to neural response are always positive (or zero), and the model incorporates the assumption that the neural activation follows (and never precedes) in time each time point of the stimulus that it is encoding. A short lag window entails that the activation of neuronal populations reflected in the MEG signal falls and rises closely following (i.e., time-locked to) the amplitude fluctuations within different frequency bands.

The procedure is repeated independently for all feature time series (e.g., for each frequency band of the stimulus spectrogram), resulting in a reconstructed time series of amplitude changes of sound spectrogram, amplitude envelope or the phoneme sequence:

(1) $${\hat{s}}_{f}\left(t\right)=\sum_{x}\sum_{\tau =\tau_{1}}^{\tau =\tau_{2}}g_{f}\left(\tau ,x\right)r\left(t-\tau ,x\right).$$

To estimate the unknown spatiotemporal response functions *g_f_*, we use the dual representation of Equation 1 and impose an L2 prior on the unknown reconstruction weights, *g_f_*(*τ*,*x*), as follows. Using matrix notation, Equation 1 can be written as *S_f_* = *RG_f_*, where we define *S_f_* ∈ ℝ^(^*^NT^*^)×1^, *G_f_* ∈ ℝ(*τx*)^×1^, and the response matrix *R* ∈ ℝ^(^*^NT^*^)×(^*^τx^*^)^, such that each row *r_n_*(*t*) in *R* represents MEG response to a spoken word or environment sound *n* across all sensors × and all time points sampled from (*t* – *τ*_2_) to (*t* – *τ*_1_). The unknown *G_f_* are estimated by minimizing the regularized sum of squared error between the original s*_f_* and the reconstructed sound features ${\hat{s}}_{f}$, e.g., spectrogram:

(2) $$\arg\;min_{G_{f}}\underset{n,t}{\sum_{n,t}{\left\{{\text{s}}_{f}\left(n,t\right)-{\hat{s}}_{f}\left(n,t\right)\right\}}^{2}}+\lambda_{f}\sum_{x,\tau }g_{f}{\left(\tau ,x\right)}^{2}.$$

Minimizing this loss function leads to the maximum-a-posteriori (MAP) estimate for *G_f_*:

$${\hat{G}}_{f}={\left(R^{\text{T}}R+\lambda_{f}\text{I}\right)}^{-1}R^{\text{T}}S_{f}.$$

This classical MAP estimate is not ideal for MEG studies where the number of conditions N are typically small compared with the dimensionality of neural responses since *R^T^ R* ∈ ℝ^(^*^τx^*^)×(^*^τx^*^)^ (Mesgarani et al., 2009; Faisal et al., 2015). Therefore, similar to kernel ridge regression (Bishop, 2006; also known as dual representation of ridge regression), we obtain the MAP estimate of the convolution model using its dual representation where the inner product *R^T^R* is replaced with corresponding Gram matrix *RR^T^*:

$${\hat{G}}_{f}=R^{\text{T}}{\left(RR^{\text{T}}+\lambda_{f}\text{I}\right)}^{-1}S_{f}.$$

To estimate the regularization parameter *λ_f_* we use a grid of predefined values for the hyperparameter and choose the optimum value that minimizes leave-one-out error within training data (Bishop, 2006). Given the lag parameters *τ*_1_ and *τ*_2_ the learned MAP estimates *g_f_*(*τ*,*x*) are used to decode the acoustic and phonetic features of a new, previously unencountered, test sound as follows:

(3) $${\hat{s}}_{f\left\{TEST\right\}}\left(t\right)=\underset{x}{\sum_{x}\sum_{\tau =\tau_{1}}^{\tau =\tau_{2}}g_{f}}\left(\tau ,x\right)r_{\left\{TEST\right\}}\left(t-\tau ,x\right).$$

The convolution model thus decodes the spectrogram at time *t* using neural responses at time (*t* – *τ*_2_),…,(*t* – *τ*_1_). To obtain an overview of the model’s performance, we used a lag window of 0–420 ms (delay from time point in the spectrogram to a range of time points in the MEG signal, i.e., from –*τ*_2_ to –*τ*_1_, where *τ*_1_ = –420 and *τ*_2_ = 0). Next, we advanced the lag window in non-overlapping 80-ms steps (20–100, 100–180,…, 340–420 ms) to investigate the dependence of the decoding accuracy on the lag timing. A relatively large step size (80 ms) was chosen due to the high computational load of the analysis and also motivated by previous research showing speech tracking at latencies of ∼100–180 ms (Abrams et al., 2008; Aiken and Picton, 2008; Koskinen et al., 2013). The same convolution model was additionally used for categorical phoneme features to investigate whether spectrogram decoding reflects the emergence of categorical phoneme information and whether the time lag differs for decoding of these two feature sets. Note, however, that the model is not optimized for categorical variables, which may affect the decoding performance.

The time-averaged frequency/modulation content of the sounds, estimated with FFT/MPS, respectively, was decoded using a regression model, in which each feature of the stimuli is reconstructed based on all time points (or a selected time window) of the MEG responses (Fig. 1*B*; Sudre et al., 2012). In the regression model, the dependent (decoded/reconstructed) variables were the semantic or non-time-varying acoustic features, while the independent variables were the MEG responses *r(t,x)* at brain location *x* and time *t* from stimulus onset. Unknown weights *w_f_*(*t*,*x*) and the L2 regularization parameters were learned in a similar fashion as for the convolution model using dual representation of the regression model (Sudre et al., 2012). Using the same notation as in Equation 1, the reconstruction value (${\hat{s}}_{f}$) for one semantic or non-time-varying acoustic feature *f* can be written as:

(4) $${\hat{s}}_{f}=\sum_{x}\sum_{t}w_{f}\left(t,x\right)r\left(t,x\right).$$

We first used MEG data at 0–1000 ms from stimulus onset to obtain an overview of model performance and then analyzed sensitivity of successive 50-ms time windows (0–50, 50–100 ms, etc.) in the MEG responses to see how the decoding of semantic/acoustic features varied with time. This resolution was considered sufficient to cover the early transient response components which were expected to be especially important for encoding the acoustic features (Salmelin, 2007). In the source space, the models were learned, for each participant, separately for the vertices within each cortical parcel, and visualized by averaging the decoding performance within each cortical parcel across participants. We visualized the sensitivity of cortical areas in decoding stimulus features by averaging the decoding performance within each cortical parcel, across all participants.

### Performance evaluation

We adopted a two-stage evaluation scheme (Palatucci et al., 2009; Sudre et al., 2012). In the first stage, the convolution or the regression model was trained to learn the unknown weights using all but two held-out items/sounds. In the second stage, the learned weights were used to decode stimulus features (acoustic, phonemic, or semantic features) for the two held-out test items. The decoding was considered correct if the combined similarity between the true features of sounds (*s*_1_ and *s*_2_) and the decoded features of the sound (*p*_1_ and *p*_2_) were greater than the reverse labeling, i.e.,

(5) $$sim\left(s_{1},p_{1}\right)+sim\left(s_{2},p_{2}\right)>sim\left(s_{1},p_{2}\right)+sim\left(s_{2},p_{1}\right),$$

where *sim*(*s*,*p*) is the Pearson correlation between original *s* and decoded features *p*. Here, all features were considered together (e.g., all frequency bands of the spectrogram). The evaluation scheme of Equation 5 was repeated for all possible combinations in a leave-two-out cross-validation approach as follows. For item-level decoding (for all acoustic and phoneme features), the 44 items (spoken words or environmental sounds) were divided into 42 training and two test sounds in all possible pair-wise combinations, leading to a total of 946 pair-wise tests. For category-level decoding (for semantic features), the two held-out test sounds were always chosen from two different semantic categories, resulting in 66 possible pair-wise tests. The evaluation was done separately for spoken words and environmental sounds. For spoken words, these test sounds were always chosen from the same speaker (i.e., within-speaker decoding); thus category-level decoding is not influenced by speaker-related acoustic differences. The reported participant-level decoding accuracy is an average over all pair-wise combinations of the held-out pairs.

### Statistical significance

Statistical significance was established by repeating each analysis with permuted data, and empirical *p* values were obtained for single participants. In each permutation run, data from one participant were chosen at random, and the item labels for the averaged evoked responses were randomly permuted across the different sounds (within spoken words or environmental sounds). This procedure was repeated 200 times for each convolution model and 1000 times for each regression model. For each permutation, the models were evaluated using all possible pairwise tests in a leave-two-out cross-validation scheme. Empirical *p* values were computed by calculating the number of times the permutation result was better than the observed decoding accuracy for each of the 16 participants. The *p* values across all 16 participants were combined using Fisher’s method and corrected for multiple comparisons (over time in the reported time courses) using false discovery rate (FDR) at an α level of 0.01. Two-tailed Wilcoxon signed-rank test was used for comparing the decoding performance between different models and sound classes across all 16 participants.

### Data and code availability

Ethical restrictions imposed by the University’s research ethics committee prevent the authors from making brain imaging data publicly available without restrictions, as these data cannot be fully anonymized. Making the data freely available under the Creative Common license would not enable us to restrict their use to scientific purposes. However, the relevant pseudonymized data used in this study are available from the authors on reasonable request and with permission of the University’s research ethics committee, for researchers aiming to reproduce the results. The machine-learning analysis has been performed with our custom-made MATLAB scripts, which are available in https://aaltoimaginglanguage.github.io/speechness/.

## Results

### Acoustic decoding of spoken words relies on neural responses that closely follow time-varying spectral features

The FFT features (non-time-varying frequency spectrum) were decoded reasonably well based on the MEG responses for spoken words using the regression model (average item-level decoding accuracy 65%, *p* < 10^−14^; Fig. 1*C*). Use of MPS and regression model resulted in similar performance (65%, *p* < 10^−11^; FFT vs MPS, two-tailed Wilcoxon signed-rank test, *Z* = 0.10, *p* = 0.94). In contrast, the time-sensitive convolution model using spectrogram was remarkably successful at decoding spoken words (83%, *p* < 10^−16^), with a significant improvement in comparison to the regression model that used time-averaged MPS (*Z* = 3.5, *p* = 0.000031; Fig. 1*C*); to obtain an overview of the convolution model’s performance, we used a lag window of 0–420 ms (delay from time point in the spectrogram to a range of time points in the MEG signal).

The success of the spectrogram convolution model in decoding spoken words was not merely due to increased information content (e.g., number of features) of the time-varying (spectrogram) compared with the time-averaged (FFT/MPS) feature sets: when we used a regression model to predict the spectrogram frequencies of spoken words by including all time-points of the MEG response to predict each time point in the spectrogram separately, the convolution model continued to perform significantly better (spectrogram regression 73%, spectrogram convolution 83%; *Z* = 3.3, *p* = 0.00031; Fig. 1*C*). Thus, the salient improvement of decoding accuracy with the spectrogram convolution model suggests that the neuronal population activity in the auditory cortices closely follows the speech signal in time, to accurately encode the minute changes within spoken words.

We then investigated whether changes in the amplitude envelope of the spoken words, corresponding to the slow temporal modulations within the speech rhythm, are important for the particularly successful decoding of spoken words, by separately decoding the amplitude envelope (spectrogram averaged across frequency) with the convolution model. The results reveal remarkably high classification performance for the spoken words (91%, significant difference from spectrogram decoding for speech, *Z* = 3.5, *p* < 0.001; Fig. 1*C*) and further highlight the importance of the temporal aspects of the stimulus for speech decoding.

### Time-locked encoding of spoken words reflects acoustic-to-phoneme mapping

Leave-one-out-reconstruction of the spoken word waveforms with the spectrogram convolution model suggested preservation of acoustic properties characteristic of different phonemes (Audio 1; Extended Data Fig. 2-1). To test this hypothesis, phonemic annotation of the speech sounds was aligned to the stimulus time course (Mesgarani et al., 2014; Di Liberto et al., 2015), and the same convolution model was used for decoding these categorical phoneme features; please note that this model is not optimized to deal with categorical variables, which may affect model performance. These phoneme sequences (Fig. 1*A*) were decoded successfully with the convolution model (73%; *p* < 10^−16^; Fig. 1*C*). Furthermore, a representation of the spoken words that combined both the speech spectrogram and the sequence of phonemes performed even above the spectrogram alone (84%; *Z* = 2.5, *p* = 0.01).

10.1523/ENEURO.0475-19.2020.audio1 Audio 1. Reconstructed audio files of a selection of spoken words and environmental sounds, based on the convolution model and spectrogram. The original stimulus is presented first, followed by the reconstructed sound; this order of presentation is used to ease the listener’s perception of relevant speech features, but please note that it produces a priming effect. For corresponding spectrograms, see Figure Extended Data 2-1. Download Audio 1, WAV file.

**Figure 2:**
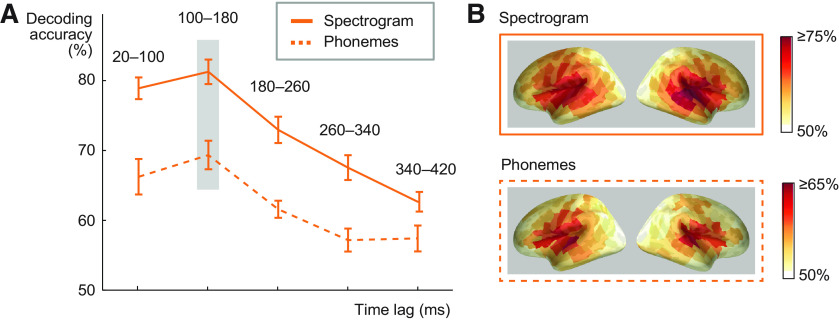
Influence of convolution lag and cortical sources for spectrogram and phoneme decoding. ***A***, Spectrogram and phoneme decoding accuracy at different lags between a time point in the stimulus and a time window in the MEG response (average across all 16 participants ± SEM). Note that the lag window does not correspond to timing relative to the stimulus onset in the MEG evoked response. The best predictive accuracy for decoding spectrogram and phoneme sequence of spoken words was reached with a lag of 100–180 ms (significant difference to 180- to 260-ms lag, *p* = 0.000031 for spectrogram, *p* = 0.016 for phonemes). ***B***, Cortical sources contributing to decoding of acoustic and phoneme features in spoken words with the convolution model at 100- to 180-ms lag (this time window showed best performance). Color scale denotes average decoding accuracy (>50%) across all 16 participants.

To determine at what delay after each time point in a sound the brain responses reflect encoding of its acoustic and phoneme information, we investigated different lag windows between time points of the stimulus and the MEG response. For this analysis, we chose the spectrogram model, which allows for a reconstruction of the spoken words that retains the relevant sound features (in contrast to the amplitude envelope alone), and additionally analyzed whether phoneme information is represented at a similar lag. The lag window was advanced in non-overlapping 80-ms steps (20–100, 100–180, and so on until 340–420 ms). Both spectrogram and phoneme decoding with the convolution model performed best when MEG responses at a lagged window of 100–180 ms after each time point in the sound were used (81% at 100–180 ms vs 73% at 180–260 ms lag *Z* = 3.5, *p* = 0.000031; for phonemes 69% at 100–180 ms vs 62% at 180–260 ms lag *Z* = 2.4, *p* = 0.016; Fig. 2*A*). For a sanity check, we also calculated the decoding accuracy for speech spectrogram using a counterintuitive lagged window of −80–0 ms, i.e., evaluating if “past” neural response could predict “future” spectrogram. With this lag, decoding performance was at chance level (mean decoding accuracy across 16 subjects 55%, SEM = 1.85). The cortical areas contributing to successful performance of the acoustic and phoneme models concentrated in and around the left and right auditory cortices (Fig. 2*B*).

To explore the contribution of semantics in the acoustic-phonetic decoding of spoken words, we investigated how these different models decoded pseudowords, by training the models with all the meaningful spoken words, and using all possible pairs of the eight pseudowords (28 combinations) for testing. Pseudoword decoding showed similar performance as decoding of the meaningful words (FFT 68%, MPS 66%, spectrogram convolution 86%, phoneme convolution 75%), with significant improvement for the spectrogram convolution compared with the MPS (*Z* = 3.4, *p* = 0.00015). The convolution model was able to decode the phoneme content of the pseudowords with a similar accuracy (75%) as for the real words. Thus, time-locked encoding does not seem to depend on lexical content, giving further support to the idea that it might reflect mapping of acoustic content to prelexical linguistic units.

### Decoding of environmental sound acoustics does not benefit from time-locked models

To investigate whether the convolution model performs better than time-averaged models for other sounds than speech, we separately applied the different acoustic models to a variety of environmental sounds (Extended Data Fig. 1-1 and Table 1-1). They conveyed the same meanings as the spoken words; thus, the conceptual endpoint of neural processing is presumed to be the same. Also, their cortical responses were similar to spoken words (Extended Data Fig. 1-2). However, the processing steps for accessing the meaning of environmental sounds are presumably different from for spoken words.

10.1523/ENEURO.0475-19.2020.f1-2 Extended Data Figure 1-2 ***A***, Sensor-level responses to spoken words (left, orange) and environmental sounds (right, blue), in one MEG sensor above the left and right temporal lobes, for one participant. The signals were averaged across 20 presentations of the same sound, from 300 ms before to 2000 ms after stimulus onset, and here also averaged over the different items within each semantic category (for visualization only). Both spoken words and environmental sounds elicited a typical time sequence of activation, with a transient response at about 100 ms that was followed by a more sustained response from about 250 ms onwards and return to baseline after 1000 ms. ***B***, Grand average (*n* = 16) dSPMs to spoken words (left) and environmental sounds (right) demonstrate that, for both types of stimuli, activation originated mainly in the bilateral temporal regions in the vicinity of the primary auditory cortex, with additional activation in inferior frontal areas, and the left hemisphere was highlighted particularly for spoken words in the later time window. Download Figure 1-2, EPS file.

**Figure 3.**
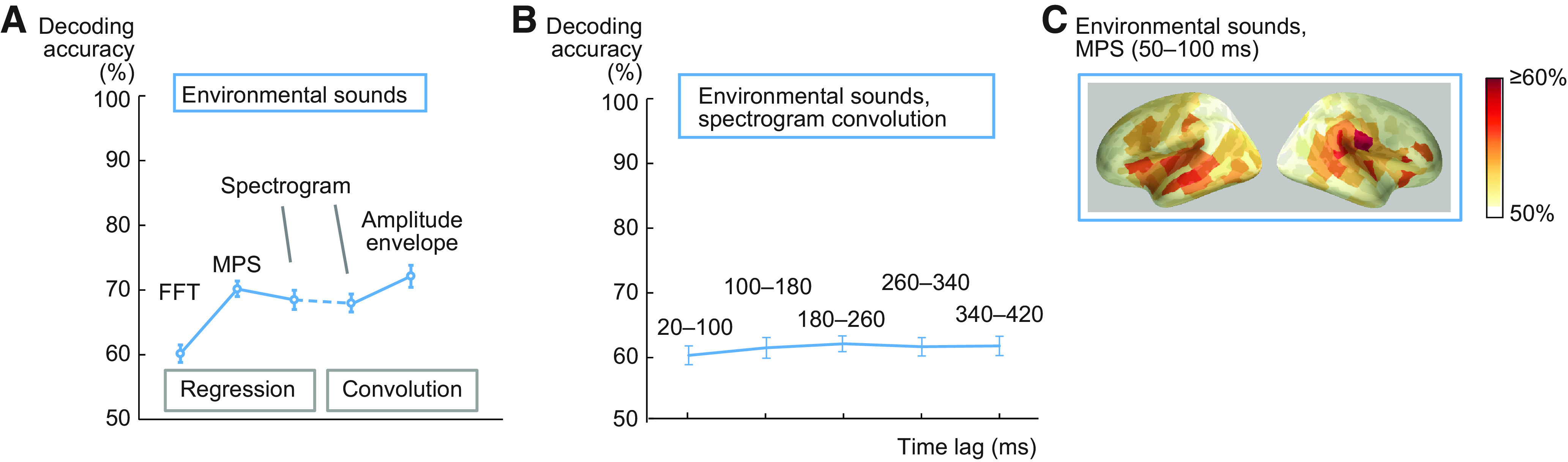
Comparison of different acoustic models for environmental sound decoding. ***A***, Predictive accuracy for environmental sounds (blue) across all test sound pairs and 16 participants (mean ± SEM). The regression model was used for the decoding of non-time-varying features (FFT frequency bins/MPS rate-scale-frequency points/semantic features), and the convolution model was used for decoding of time-varying features (spectrogram time-frequency points/amplitude envelope); for a control analysis, the spectrogram was also decoded with the regression model. ***B***, Investigation of convolution lag on spectrogram decoding accuracy for environmental sounds (average across all 16 participants ± SEM). ***C***, Cortical sources contributing to decoding of acoustic features in environmental sounds with the MPS regression model at 50–100 ms after stimulus onset (performance was best with this model and time window). Color scale denotes average decoding accuracy (>50%) across all 16 participants.

The FFT features were decoded based on the MEG responses for environmental sounds at a significant level with the regression model (60%, *p* < 0.0001; Fig. 3*A*). Use of MPS together with regression model resulted in better performance than FFT (70%, *p* < 10–15; FFT vs MPS, *Z* = 3.5, *p* = 0.000031); such an improvement in decoding was not observed for spoken words (for spoken words FFT and MPS decoding were both at 65%). In contrast, the accuracy with spectrogram convolution (68%, *p* < 10^−4^) did not improve compared with the MPS result (*Z* = 1.1, *p* = 0.27), as was observed for words (spectrogram convolution decoding for words was at 83%, with significant difference between spoken words and environmental sounds: *Z* = 3.5, *p* < 0.0001). Using a regression model to decode the spectrogram frequencies of environmental sounds resulted in similar decoding performance (68%, *Z* = 0.18, *p* = 0.87) and was also fairly similar to spoken word decoding (spectrogram regression 73%; no significant difference between spoken words and environmental sounds: *Z* = 1.9, *p* = 0.058). No time dependence was observed for environmental sounds using different lag windows (61% at 100–180 ms vs 62% at 180–260 ms lag; *Z* = 0.052, *p* = 0.98; Fig. 3*B*). The amplitude envelope decoding showed some improvement for the environmental sounds compared with the spectrogram convolution decoding (*Z* = 2.3, *p* = 0.021), but was still low (72%) compared with the remarkably high decoding performance for speech amplitude envelope (91%; significant difference between spoken words and environmental sounds: *Z* = 3.5, *p* < 0.0001). Similarly to the convolution spectrogram decoding for speech, the cortical areas contributing to decoding environmental sound acoustics mainly concentrated in and around the left and right auditory cortices (Fig. 3*C*).

### Enhanced performance with the time-locked model for spoken words, but not environmental sounds, is not explained by possible confounds in the comparison of models

In visual inspection, all sounds showed typical auditory evoked responses at the sensor level, at good SNR (Extended Data Fig. 1-2*A*), with activation around the auditory cortices in the left and right temporal lobes (Extended Data Fig. 1-2*B*). Subtle differences in the averaged evoked responses between spoken words and environmental sounds were not investigated; only the variability among items (within spoken words or environmental sounds) affects the machine learning modeling.

However, as the sounds were a selection of natural sounds, there were several differences between the spoken words versus environmental sounds (Extended Data Figs. 3-1*A*, 3-2). Thus, we sought to rule out potential confounds arising from these differences that could be thought to influence the observed striking improvement of decoding performance with the convolution model for speech but lack of similar improvement for environmental sounds.

The benefit for spoken word but not environmental sound decoding was not due to larger dissimilarity among the spoken word stimuli with added temporal detail: while the spectrograms of spoken words do show larger dissimilarity than the MPSs (two-tailed *t* test: *t*_(945)_ = 139.5, *p* < 0.001), this is also true for the environmental sounds (*t*_(945)_ = 14.8, *p* < 0.001; Extended Data Fig. 3-1*B*). Notably, the dissimilarity among items, which typically leads to better decoding performance, is overall significantly greater for the environmental than speech stimuli, also with the spectrogram model (*t*_(945)_ = 22.5, *p* < 0.001). This confirms that increased stimulus dissimilarity alone cannot explain the significantly better classification performance for spoken words than environmental sounds.

We also verified that the difference for time-locked decoding between spoken words and environmental sounds was present already at the onset of the stimulus: The spectrograms were cut to 0–100 ms in length and decoded with the convolution model using a lag window of 100–180 ms. This resulted in an average decoding accuracy of 69% for spoken words and 58% for environmental sounds (significantly better for spoken words; *Z* = 3.16, *p* = 0.002). Thus, although the decoding performance is overall worse with only the start of the word/sounds, as we expected, a significant difference remains between the spoken words and the environmental sounds (for the whole duration of the words and the same lag the decoding accuracies were 81% for speech and 61% for non-speech). This additional analysis shows that the enhanced decoding of words with the time-locked model is not explained by longer analysis time that would be needed to extract word meanings; instead, the time-locked model applies from the start of the word.

Furthermore, the benefits of the convolution model for modeling the cortical encoding of spoken words are not restricted to the leave-two-out classification task: direct leave-one-out reconstruction (correlation of the original and reconstructed features) demonstrated that spoken words were better reconstructed with the spectrogram convolution than MPS regression model (0.19 vs 0.10, *Z* = 3.5, *p* < 0.001; Extended Data Fig. 3-1*C*; for examples of reconstructed sounds, see Extended Data Fig. 2-1, Audio 1), whereas environmental sounds were better reconstructed with the MPS regression than spectrogram convolution (0.14 vs 0.08, *Z* = 3.5, *p* = 0.001; Extended Data Fig. 3-1*C*; for examples of reconstructions based on the spectrogram convolution model, see Extended Data Fig. 2-1, Audio 2). The amplitude envelope of spoken words was also remarkably well reconstructed in comparison to the amplitude envelope of environmental sounds (reconstruction accuracy for spoken words 0.67 and for environmental sounds 0.32; *Z* = 3.5, *p* < 0.001; Extended Data Fig. 3-1*C*). Moreover, correlating the pair-wise reconstructed spectrogram distances with the distances between original sound spectrograms shows that relatively more information is preserved in the reconstructions of spoken words compared with environmental sounds based on their respective cortical responses (Spearman correlation *r *=* *0.41, *p* < 0.001 for spoken words, *r *=* *0.22, *p* < 0.001 for environmental sounds; Extended Data Fig. 3-1*D*).

10.1523/ENEURO.0475-19.2020.audio2 Audio 2. Reconstructed audio files of a selection of environmental sounds, based on the convolution model and spectrogram. The original stimulus is presented first, followed by the reconstructed sound. For corresponding spectrograms, see Extended Data Figure 2-1. Download Audio 2, WAV file.

10.1523/ENEURO.0475-19.2020.f2-1 Extended Data Figure 2-1 Original and reconstructed spectrograms of selected spoken words (top) and environmental sounds (bottom) based on the convolution model. The corresponding sound files are in Audios 1, 2. Download Figure 2-1, EPS file.

10.1523/ENEURO.0475-19.2020.f3-1 Extended Data Figure 3-1 ***A***, The median (with 25% and 75% interquartile ranges) power spectra (left) as well as spectral scales (middle) and temporal rates (right) pooled across the 128 frequency bins, for speech and environmental stimuli, illustrating the differences between spoken words and environmental sounds. Also, the environmental sounds have more variability in their spectral scales and temporal rates, whereas spoken words display prominent slow temporal modulations. ***B***, Pair-wise distances (1 minus correlation) of the original stimulus MPS and spectrogram (mean ± SD). ***C***, Leave-one-out reconstruction fidelity, i.e. correlations of reconstructed and original MPSs/spectrograms/amplitude envelopes for spoken words and environmental sounds (mean ± SEM). ***D***, Scatterplot of the pair-wise distances (1 minus correlation) among original (*x*-axis) and pair-wise distances among reconstructed (*y*-axis) spectrograms. Download Figure 3-1, EPS file.

10.1523/ENEURO.0475-19.2020.f3-2 Extended Data Figure 3-2 The median (with 25% and 75% interquartile ranges) power spectra (left), as well as spectral scales (middle) and temporal rates (right) pooled across the 128 frequency bins, for speech and different categories of environmental stimuli. The human non-speech sounds are most similar to speech in the spectral scales and temporal rates. Download Figure 3-2, EPS file.

**Figure 4.**
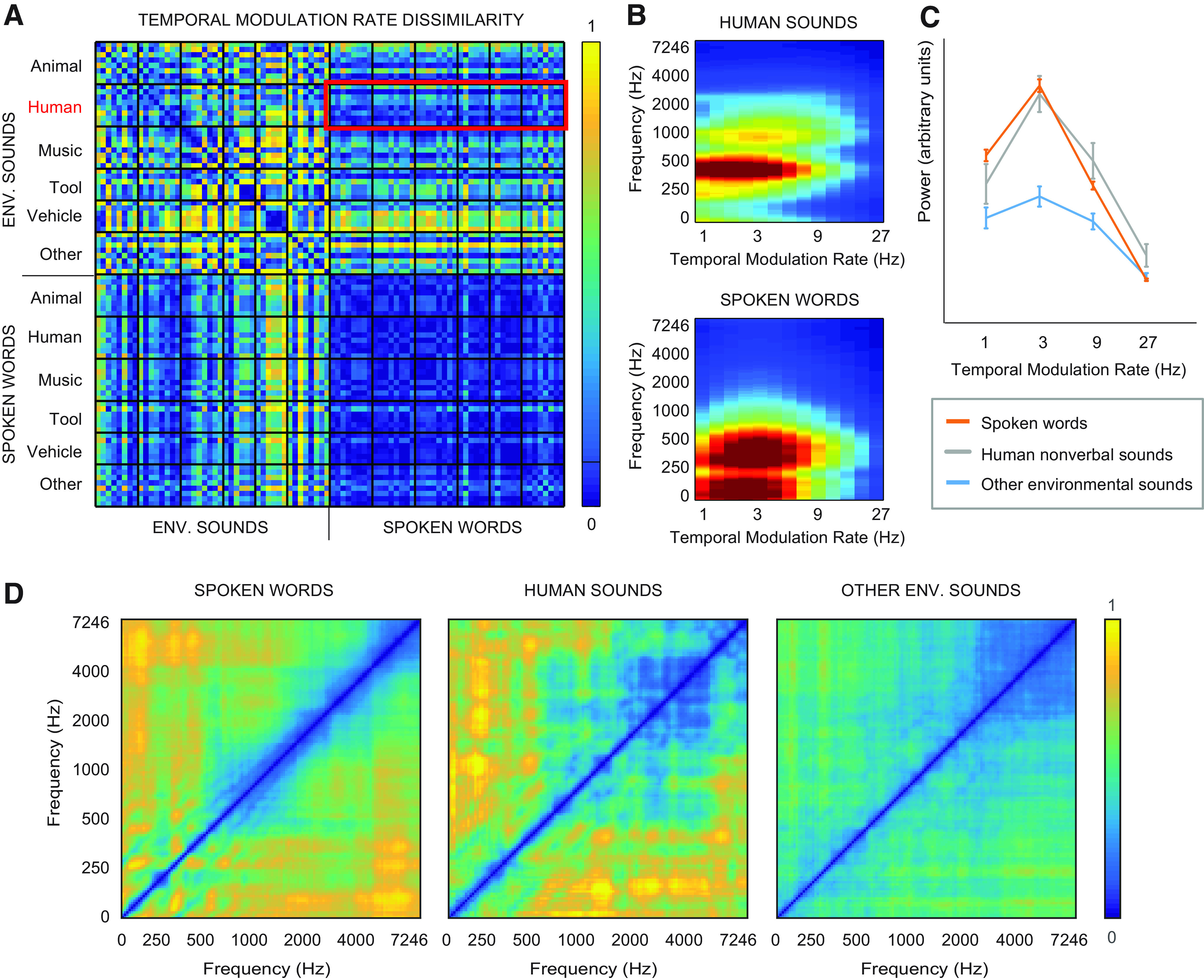
Comparison of human nonverbal sounds and spoken words. ***A***, Dissimilarity (1 minus correlation) of the MPS temporal rates across the environmental sounds and spoken words. ***B***, Average temporal modulation rate × frequency representations for human sounds (top) and spoken words (bottom). ***C***, Power (mean ± SEM) at different temporal modulation rates for spoken words (red), human nonverbal sounds (gray) and other categories of environmental sounds (blue). ***D***, Dissimilarity of different frequency bands of the sounds, indicating the degree of correlated temporal modulations (co-modulations), calculated separately for each item, and averaged over spoken words (left), human nonverbal sounds (middle), and other environmental sounds (right).

The improved performance of the time-locked encoding model for spoken words was also unlikely to be solely due to different kinds of temporal properties of the words and other sounds. Specifically, speech has prominent slow (1–7 Hz) temporal modulations (Fig. 4*A*,*B*; Extended Data Figs. 3-1*A*, 3-2) that are important for its intelligibility (Elliott and Theunissen, 2009) and have been suggested to be represented in the auditory cortices through a linear coding scheme (Pasley et al., 2012). Environmental sounds produced by the human vocal tract (laughter, crying, etc.) are very similar to speech in terms of spectrotemporal characteristics and temporal modulation rates (with a small difference observed only for the highest rate (*t*_(50)_ = 4.5, *p* = 0.004; Bonferroni corrected for multiple comparisons; Fig. 4*C*). Spoken words showed a more variable spectral structure across time (larger values of SSI) than environmental sounds (*t*_(86)_ = 3.9, *p* < 0.001). However, the SSI did not differ between spoken words and human nonverbal sounds (*t*_(50)_ = 0.45, *p* = 0.65), which were similar to each other also in their co-modulation properties (correlated temporal modulations between frequency channels; Fig. 4*D*). Yet, we saw no improvement in decoding of this subset of environmental sounds with the convolution model (MPS regression 68% vs spectrogram convolution 69%; *Z* = 0.31, *p* = 0.84). The results indicate that particularly spoken words, with their specific combination of spectrotemporal features, show remarkably improved decoding when neuronal population activity in the auditory cortices is modeled as tightly following the time-evolving acoustics.

### Semantic features are successfully decoded for both spoken words and environmental sounds, but with different timing

To ensure that the sounds were processed up to their meaning, the participants’ task was to identify immediate repetitions of the same meaning, i.e., two different exemplars of a sound presented one after the other. The task was easier for the spoken words than for the environmental sounds (average percentage of hits for spoken words 94%, for environmental sounds 78%; two-tailed Wilcoxon signed-rank test, *Z* = 3.6, *p* = 0.00003). Reaction times (RTs) were also longer for the environmental sounds (average RT for hits 1.7 s) than the spoken words (average RT for hits 1.5 s; two-tailed Wilcoxon signed-rank test, *Z* = 3.5, *p* = 0.001). The same set of semantic features (Fig. 1*A*, right) was used for modeling the environmental sounds and spoken words. The semantic feature representations captured meanings of individual items and formed salient clusters of the five semantic stimulus categories. We focused on telling apart two sounds from two different categories based on their reconstructed features (combination of question features and text corpus features). The regression model was successful in decoding the semantic category of both environmental sounds (81%; *p* < 10^−15^) and spoken words (58%; *p* = 0.0015), with significantly better accuracy for environmental sounds (*Z* = 3.5; *p* = 0.000031).

**Figure 5.**
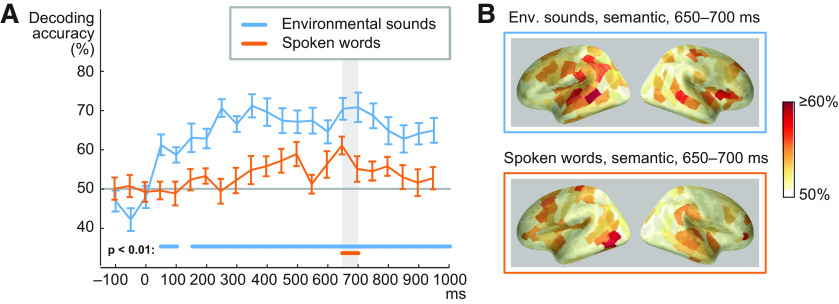
Comparison of semantic decoding for spoken words and environmental sounds. ***A***, Time course of regression-model based predictive accuracy in semantic features of environmental sounds and spoken words (16 participants, mean ± SEM). The values on the *x*-axis represent the starting time of the 50-ms time windows in the MEG responses. Time 0 indicates the onset of the sound stimulus. Decoding of the semantic feature set was performed for successive 50-ms time windows for the whole stimulus duration, for both stimulus types. The time windows with statistically significant decoding (*p* < 0.01) are indicated by thick horizontal lines above the *x*-axis. Gray solid line denotes chance level performance (50%). The gray bar represents the time window used for source level decoding. ***B***, Source areas contributing to decoding of semantic features for environmental sounds and spoken words at 650–700 ms from stimulus onset. This time window had significant decoding performance for both classes of sounds.

Semantic decoding reached significance for environmental sounds at 50–100 ms and remained significant (*p* < 0.01) from 150 ms on until the end of the analysis window (Fig. 5*A*); we analyzed sensitivity of all successive 50-ms time windows in the MEG responses. In the same analysis for spoken words, best performance for decoding spoken word semantics was reached late and was significant only at 650–700 ms (Fig. 5*A*). In contrast, MPS-based acoustic decoding of environmental sounds was significant at 50–150 and 200–300 ms after stimulus onset for environmental sounds and at 250–300 ms for spoken words. Cortical sources contributing to semantic decoding are illustrated in Figure 5*B* (see also Extended Data Fig. 5-1).

10.1523/ENEURO.0475-19.2020.f5-1 Extended Data Figure 5-1 Illustration of the cortical parcellation template and the top 20 ranking cortical areas (parcels) for the decoding of acoustic (orange) and semantic (red) features for spoken words (left), and acoustic (light blue), and semantic (dark blue) features for environmental sounds (right). The parcel names are listed in the table below. Sources for acoustic decoding of spoken words are based on the convolution model and spectrogram, with the best lag (100–180 ms). Sources for acoustic decoding of environmental sounds are based on the regression model and MPS, at 50–100 ms. For semantic decoding, the regression model at 650–700 ms was used for both classes of sounds. L = left, R = right. Download Figure 5-1, EPS file.

## Discussion

### Cortical activation faithfully tracks the spectrotemporal detail of spoken words

The cortical processing of speech as compared with the processing of other sounds has remained a major open question in human neuroscience. Here, by combining time-sensitive brain imaging and advanced computational modeling, we discovered that the acoustic-phonetic content of natural, meaningful spoken words is encoded in a special manner, where the cortical evoked responses faithfully track in time the unfolding spectrotemporal structure and the amplitude envelope of the spoken words. This time-locked encoding was observed also for meaningless pseudowords. However, responses to environmental sounds, even human-made non-speech sounds with spectral and temporal modulations comparable to speech, did not show improved decoding with the dynamic time-locked mechanism, and were better reconstructed using the time-averaged spectral and temporal modulation content, suggesting that a time-averaged analysis is sufficient to reach their meanings.

Phase-locking of oscillatory activation to the amplitude envelope of sounds has recently gained wide interest as a potential mechanism for cortical encoding of speech (Ding and Simon, 2014; Ding et al., 2016). However, it has remained controversial whether entrainment is directly related to speech parsing: it might reflect several different underlying processes (Ding and Simon, 2014), some of which are not specific to speech nor to the human auditory system (Steinschneider et al., 2013). Based on the current results, cortical auditory evoked activation tracks fine-scale spectral detail of single spoken words in a temporally accurate manner, relying heavily on both the spectrotemporal and overall amplitude changes within the sound. We propose that this time-locking of evoked responses to an unfolding spoken word may partly reflect the same phenomenon as the reported entrainment of oscillatory activity to continuous speech envelope. It has been suggested that phase-locking of oscillatory activation to the stimulus amplitude envelope may be driven by acoustic edges at syllable onsets (Doelling et al., 2014), and it can be modeled as a series of transient responses to changes in the stimulus (Aiken and Picton, 2008). Furthermore, the spectral content and temporal modulations within speech are non-independent, and envelope entrainment also depends on fine spectrotemporal detail (Ding et al., 2014). Thus, phase-locked oscillations can at least partly be thought of as reflecting the superposition of transient evoked responses that track the fine-scale spectrotemporal evolution of the speech stimulus (Alexandrou et al., 2018; Obleser and Kayser, 2019); this does not exclude the possible involvement of endogenous oscillatory activity. Here, for evoked responses to isolated spoken words the best decoding performance was obtained for the amplitude envelope of the sound (reconstructed with 67% accuracy), indicating that the cortical responses carry a lot of the characteristics of the envelope. However, the particularly high performance for the amplitude envelope compared with the spectrogram may be partly due to the reduced dimensionality of the decoded features (1 amplitude envelope vs 128 frequency bands, respectively), reducing the influence of noise. Also, the different acoustic features within speech are temporally coupled, and the online analysis of features in the spectrotemporal fine structure is modulated by cues in the envelope (Shamma et al., 2011). As an average of all the frequency channels, the amplitude envelope may carry the most prominent temporal events in the spoken words, and its decoding may thus highlight the most salient speech-tracking features of the MEG signal.

Speech comprehension has been shown to rely strongly on slow-rate temporal modulations of the speech envelope (Elliott and Theunissen, 2009). Notably, in our study, human vocalizations, the environmental sounds most similar to speech in that they contain prominent slow temporal modulations as well as similar co-modulations across frequencies, did not show any improvement in decoding with the time-sensitive spectrogram model. The dynamic mode of encoding uncovered here thus seems to take place for the particular combination of spectrotemporal features that are characteristic of speech. The high performance (83%) of the spectrogram convolution model, in which each time-frequency-point was reconstructed based on the MEG signal, showed that the fine spectral content of speech is tracked alongside the amplitude envelope at a high temporal resolution, and the spoken word spectrograms can be fairly accurately reconstructed based on this model. Furthermore, the difference in decoding performance is visible right form the onset of the sound, for the first 100 ms, when the sound is first recognized as speech. Our results, along with recent findings based on decoding sounds from fMRI responses (Santoro et al., 2017), indicate that human auditory cortical processing might be optimally tuned for encoding the spectrotemporal structure of speech.

The current results corroborate previous work highlighting the importance of temporal modulations within speech (Overath et al., 2015), but they should not be interpreted to mean that auditory cortical activation would not be time-locked to non-speech sounds. Non-speech sounds also elicit prominent cortical auditory evoked responses time-locked to the acoustic changes within the sound, and phase-locking of cortical oscillations to the amplitude envelope is observed also for non-speech sounds (Doelling and Poeppel, 2015). The current results further show that the amplitude envelope of environmental sounds is tracked in cortical evoked responses, as it can be reconstructed at 32% accuracy with the convolution model. Thus, a part of the time-locking phenomenon seems not to be specific to speech. However, overall, the best reconstruction of the environmental sounds (14%) was achieved by modeling their time-averaged modulation content (MPS), suggesting that the time-averaged spectral and temporal modulation content may be the information most accurately represented in cortical activation and sufficient for accessing meanings of non-speech sounds. Indeed, the identification of common sounds in the environment may be based on the encoded summary statistics (McDermott et al., 2013; Santoro et al., 2014). Interestingly, the overall modulation content was a more accurate model of environmental sounds encoding than the frequency spectrum alone, whereas for speech there was no such improvement with modulation filters, consistent with a recent fMRI study (de Heer et al., 2017).

For mapping speech acoustics to linguistic representations, accurate dynamic encoding of the temporal evolution within each frequency band at each time point is needed. An interesting parallel can be found from animal studies, where single-unit recordings in the auditory cortex suggest frequency-bin-selective synchronization of neuronal population discharges to the temporal envelope of species-specific calls, which in many ways resemble human speech; thus, temporal envelope information within different frequency bands in behaviorally relevant vocalizations might be encoded cortically by coherent discharge patterns in distributed neuronal populations (Gehr et al., 2000; Nagarajan et al., 2002; Gourévitch and Eggermont, 2007). The current results suggest that, in humans, similar encoding mechanisms might have become especially important for speech, with extensive exposure to the language environment during development. Future studies should determine if similar time-locking might be observed with tasks requiring attention to fine-grained temporal detail or specialization to categorical perception in other sounds through experience, e.g., for instrumental sounds in musicians.

### Accurate temporal encoding of the acoustic stream is necessary for lexical and semantic access of spoken words

Despite the different acoustic content of spoken words and corresponding environmental sounds, their processing converged at the endpoint; the same set of semantic features was successful in decoding both classes of sounds. Spoken words showed relatively low semantic decoding performance, in line with previous studies (Simanova et al., 2010; Correia et al., 2015). One reason for the different semantic decoding performance of spoken words and environmental sounds may have been the fact that environmental sounds are inherently less familiar and their semantic access may thus require a more active effort and longer time than for spoken words. This conclusion is also suggested by the performance in the one-back task (detecting two subsequent sounds with the same meaning), which was more effortful, with more errors and longer reaction times, for environmental sounds than spoken words. Nonetheless, our set of environmental sounds was tested for fast and consistent naming. Together, results of the behavioral task and the semantic decoding results indicate that attentional engagement was equal or even stronger for the environmental sounds than spoken words, and in fact more effort and time was needed to decipher the meanings of the environmental sounds. Thus, increased attention or longer identification times cannot explain the strikingly more successful acoustic decoding for spoken words than environmental sounds. Furthermore, the differential decoding performance was visible already during the first 100 ms of the sounds.

Semantic decoding of environmental sounds first reached significance at 50–100 ms, at the same time as acoustic decoding, and continued to be successful until the end of the analysis period, whereas semantic decoding of spoken words reached significance at 650–700 ms (this one significant time bin likely represents only a part of a wider time range). The current results are complementary to traditional experimental paradigms of semantic processing, where the violation of semantic expectation for spoken words and sounds shows in cortical responses at around 200–700 ms (Aramaki et al., 2010; Frey et al., 2014; Hendrickson et al., 2015). Comparable latencies (200–700 ms) were also recently observed in decoding the similarity of spoken word meanings based on preceding content in continuous speech, using EEG (Broderick et al., 2018). The current decoding results indicate that for environmental sounds the acoustic features may be informative of the physical sources (i.e., meaning) of the sound early on, but that the identification may be extended in time or its timing and duration may be variable between sounds, whereas for spoken words identification seems to occur within a more distinct and narrow time window, when the correct word candidate has been identified in the lexicon. The identification of spoken words involves the comparison of the incoming input signal to the lexical representations stored in memory in a continuous manner and selection of the correct lexical candidate (Marslen-Wilson, 1987). For our stimulus words, which contained compound words, the uniqueness point and selection of one lexical candidate for semantic access was fairly late (on average 500 ms), which may have contributed to the late time window for the semantic decoding.

Both phoneme and acoustic information could be decoded from the MEG signal best at a latency of 100–180 ms, suggesting that those representations might exist simultaneously in the cortical activation sequence. It has to be noted, that our decoding model was not optimally designed for decoding categorical variables such as the phoneme labels which probably explains the overall lower decoding accuracy for phoneme labels (73%). However, the similarity of the optimal lags in spectrogram and phoneme decoding is not affected by this confound. Several other studies also indicate that the optimal temporal integration window for parsing speech acoustics into linguistic units might lie within this range. The amplitude envelope of continuous speech is tracked by phase-locked cortical oscillations at 100- to 180-ms latency (Abrams et al., 2008; Aiken and Picton, 2008; Koskinen et al., 2013), and this has also been shown to be approximately the time frame in which the categorical neural organization for phonemes transiently emerges (Chang et al., 2010). Also, studies with isolated speech syllables have demonstrated that the evoked responses can be seen as a combination of transient “impulse responses” to the onsets of constituent phonemes, with latencies of ∼100–200 ms (Ostroff et al., 1998; Tremblay et al., 2003). However, the performance of the convolution model for spectrogram and phoneme decoding was fairly high also at 20- to 100-ms lag, suggesting that the acoustic-phonetic content of the words might in fact be tracked over multiple different integration windows. This may reflect the encoding of phonemes with different temporal characteristics (Khalighinejad et al., 2017), but may also be related to simultaneous phoneme and syllable level encoding, similarly to what has been suggested in entrainment of nested cortical oscillations (Ding and Simon, 2014; Ding et al., 2016). However, obtaining temporally and spatially distinct neural response fields for each phoneme was not the goal of this study, and forward models would be more useful for this purpose.

Categorical perception of phonemes is a well-established phenomenon in behavioral studies, and its development is the basis of tuning to mother tongue in early infancy. However, the neural underpinnings of this phenomenon are still debated. Previous neuroimaging studies have shown that the superior temporal areas are tuned to phoneme categories, which rely on integration of several spectral and temporal cues (Formisano et al., 2008; Mesgarani et al., 2014). In the current results, spoken word decoding improved, compared with the spectrogram, when we included a combination of the spectrogram and the phoneme sequence of the word. These results echo those of a recent EEG study, where articulatory features of phonemes and the spectrogram together were the best model for decoding continuous speech based on phase-locked cortical oscillatory activation (Di Liberto et al., 2015). These results together might be interpreted to suggest pre-lexical categorical representations that are separate from the analysis of acoustic properties of the speech signal. However, a recent MEG study (Daube et al., 2019) suggested that the gain in predictive power of the phonemic/articulatory features may be explained by acoustic features of the phonemes: When including Gabor patterns of the sounds (different spectrotemporal modulations akin to our MPS model, with a time series) or even only the phoneme onset timings, performance gain was similar as when the categorical articulatory features were combined with the spectrogram. In that study, the best model was a relatively simple acoustic feature space that focused on acoustic edges. This might suggest that purely acoustic models are sufficient in explaining MEG/EEG responses to speech. Thus, it remains unclear what the successful decoding of categorical phonemes or phonemic features across studies tells us about prelexical stages of speech processing, and further research is needed.

In any case, the current results are compatible with the idea that linguistic representations emerge directly from tuning to the complex spectrotemporal acoustic features characteristic of different phonemes (Mesgarani et al., 2014; de Heer et al., 2017) and that the quasi-rhythmic changes in the amplitude envelope at syllable boundaries might be crucial in guiding this process. As noted above, the processing step where the continuous acoustic signal interfaces with phonological representations is only required for speech, and this is where time-locked encoding for speech appears to come into play. Decoding performance was similar for pseudowords and real words, indicating that this mode of encoding is related to the low-level parsing of the acoustic signal, and might not be directly affected by the lexical-semantic status of the speech utterance. However, this warrants further examination. Speech processing is interactive, such that top-down influences from semantic and syntactic context are expected to influence low-level acoustic processing, particularly with sentence-level and narrative speech. Indeed, a recent fMRI study showed that not only the spectral content and phonemic features, but also semantic features within narrative speech are represented in highly overlapping regions early in the acoustic processing hierarchy (de Heer et al., 2017), indicating top-down influences of semantic content on acoustic and phonological processing. In the current study, cortical sources contributing to acoustic and phoneme decoding were both concentrated around bilateral auditory cortices and did not show clear lateralization, consistent with previous studies (de Heer et al., 2017; Brodbeck et al., 2018) and compatible with the view that acoustic-phonetic processing is bilaterally implemented (Poeppel, 2003).

### Future directions

The present finding of time-locked encoding for speech, but not other sounds, deepens the understanding of the computations required for mapping between acoustic and linguistic representations. The current findings raise the question of what specific aspects within sounds are crucial for cueing the brain into using this special mode of encoding. Future work could investigate the contribution of different statistical properties within speech acoustics by using synthetized stimuli, the possible effect of experimental task to boost the use of time-locked or time-averaged mode in sound processing, and the role of top-down semantic contributions using real-life like auditory environments. The rapidly computable convolution model for high-dimensional MEG/EEG signals can be further developed for decoding of a time-varying modulation representation, which might even better model the time-locked cortical encoding of speech (Pasley et al., 2012). Finally, the present findings may in the future help to bridge the gap between investigations of cortical temporal tracking of continuous speech and isolated linguistic stimuli.

## Synthesis

Reviewing Editor: Satu Palva, University of Helsinki Decisions are customarily a result of the Reviewing Editor and the peer reviewers coming together and discussing their recommendations until a consensus is reached. When revisions are invited, a fact-based synthesis statement explaining their decision and outlining what is needed to prepare a revision will be listed below. The following reviewer(s) agreed to reveal their identity: Giovanni Di Liberto.

This is a well-designed MEG study that measures how strongly the MEG signal reflects sound stimuli that were either words or corresponding environmental sounds. Different decoding models were employed and compared. Main message of the manuscript is that spoken words can be better classified using a convolution model, which includes time-varying features of the signal, whereas environmental sounds can be better classified using a regression model, which does not include time-varying features. This is taken as evidence for two different processing modes in response to speech and other sounds, respectively.

In addition, the authors looked at various acoustic and higher-level speech representations, adding relevant and interesting results to the current literature on this topic.

Major

1. The interpretation that our brains use different processing modes for speech vs other sounds is very interesting, but I wonder whether this could be a trivial consequence of the used stimuli. It seems that environmental sounds can be identified or “comprehended” very early on (the semantic decoding analysis suggests after ∼ 50 to 100 ms), whereas words need to be analysed for a longer time to extract their meaning (the semantic decoding analysis suggests after 650 to 700 ms, and the uniqueness point is on average at 500 ms). Of course, the semantic decoding analysis cannot exactly reveal how much of the stimulus is needed to understand it, but it can serve as a proxy. This means that words need to be analysed ∼450-600 ms longer before a meaning can be extracted, thus the temporal tracking is necessary for a longer time because it adds new information. For other sounds no new information is inherent in the stimulus after the initial analysis of the sound. This would also mean, if environmental sounds were used that could only be identified after the same time as spoken words, the convolution model would be just as useful. In other words, there might well be two different modes, but they are not employed differently between words and other sounds, but they are employed depending on whether they are useful.

It is tricky to rule out this trivial explanation with the current stimuli, but one way I can think of is to repeat the analysis with a shorter time window (e.g. the first 100 ms of the words/sounds). With this shorter time window, the increased decoding performance from regression to convolution model should disappear (and if it is still there, and still not present for sounds, this would make a strong case for the author’s interpretation). Please report the results of this analysis and their interpretation in the reply and potentially in the manuscript.

2. A second finding that the authors highlight is that “Time-locked encoding of spoken words reflects acoustic-to-phoneme mapping” (line 497). The underlying rationale is the finding by DiLiberto et al 2015. There have been some important developments regarding this finding in: Daube, C., Ince, R. A., & Gross, J. (2019). Simple acoustic features can explain phoneme-based predictions of cortical responses to speech. Current Biology, 29(12), 1924-1937. This study shows that phoneme-locking does not necessarily reflect a linguistic representation of phonemes, but is based entirely on acoustic features (i.e. the phoneme structure of speech is contained in the acoustic signal, and does not need to be “mapped” or “extracted”). The manuscript should include this development and discuss the implications in the discussion section.

3. The concepts of oscillations, entrainment and evoked responses are mixed up throughout the manuscript. Most notably this is seen on page 23, line 677: “phase-locked oscillations can be thought of as reflecting the superposition of transient evoked responses”. There is a current debate about the contribution of endogenous oscillations (which could be entrained by speech) versus only evoked responses (which occur to ANY sound). Evoked responses are not related to oscillations, and a time-locked mechanism does not “account for observations of oscillatory entrainment” (line 673). This distinction should be made clearer throughout.

4) The authors use a backward modelling analysis method, or decoding model, that allows to reconstruct a stimulus vector given the corresponding neural data. Specifically, the authors refer to this method as “regression” or “convolution” while, in fact, the method is just one. It’s a linear regression that, in some cases, uses a convolutional kernel that includes various time-latencies. However, the authors compare results for continuous and categorical vectors (e.g. speech spectrogram and phonemes), which is problematic as the model is tailored to continuous variables. Other approaches (e.g. logistic regression) would be better suited for this application. In fact, the authors measured much lower “prediction” scores for categorical (phonemes and semantics) than continuous variables. However, they don’t seem to consider the method itself as a potential cause for this result. This is an issue, as part of the discussion is centred on this result. Similarly, the manuscript could be clearer on how exactly the “prediction” scores were calculated for categorical variables.

5) Why was the decoding analysis done in sensor space instead of source space?

6) The modelling approach has at least other two issues that could be easily solved and should be discussed. First, the regression (“convolution model”) constitutes a backward model fit that decodes a stimulus vector given the corresponding MEG signal. However, the mathematical formulation indicates that the reconstruction is performed on each feature independently (e.g., phoneme A, phoneme B; multivariate-to-univariate), which is particularly problematic for sparse categorical variable. Approaches such as Canonical Correlation Analysis (for example, see the work from Alain de Cheveigné’s lab and Lucas Parra’s lab) would solve this issue as they are multivariate-to-multivariate mapping methods. This issue should be discussed as it can also explain why the results for the envelope are much better than the ones for the phoneme descriptor. The second issue regards the study of the temporal latencies of the effects. While the approach adopted by the authors is valid, it is certainly suboptimal. A more reasonable approach would be, instead, to reverse the regression approach to fit forward models, which are instead directly physiologically interpretable and take into account all stimulus features and all time-latencies at the same time. Note that backward models can also be mathematically transformed into the corresponding forward models (Haufe et al., NeuroImage, 2014).

7) Based on my previous comments, several discussion points should be toned-down. The current analysis has issues that may undermine some of those interpretations.

.

Minor

The study had only 16 participants (and no a priori estimated sample size), which is becoming less and less acceptable in psychological and neuroimaging studies (e.g. Simmons JP, Nelson LD, Simonsohn U (2011) False-positive psychology: Undisclosed flexibility in data collection and analysis allows presenting anything as significant. Psychological science 22: 1359-1366. https://doi.org/10.1177/0956797611417632). While it would be unfair to request additional data, a sample size of 16 is not something that should be advocated, particularly in a wide-reaching journal like eNeuro. Please add an explicit justification for this low number of participants and demonstrate awareness that this is not good scientific practice On page 21, line 613, there appears to be an randomly chose p-value (“Bonferroni corrected significance limit 0.0125”). Please justify this based on similar previous analyses.

Which programme/software was used for the decoding analyses?

The conclusion starts with “The present discovery of a dynamic, time-locked mechanism for speech processing”. The faithful, “time-locked” tracking of speech and other sounds is well-established and I wouldn’t go as far as calling it a discovery. The thorough comparison between speech and other sounds is the novel aspect of this study, in my opinion.

The final sentence of the conclusion (page 28, line 818) states: “the present findings offer a crucial link between investigations of cortical processing of continuous speech and isolated linguistic stimuli”. How does this study offer a link between both when it only addresses isolated words and sounds?

Line 75: “His” -> “This”?

Line 81: Those studies showed that neural signals reflect phoneme responses, true. However, saying that phonemes have been successfully decoded sounds like an overstatement if not clarified. “Successfully decoded” could sound as if can already build a brain-to-speech device, while that’s not true, not yet. Please clarify.

Lines 82-84: This is a very important point that clarifies the novelty of this work. I feel that this sentence can be improved to make your point more strongly. For example, “all of these levels of analysis”: You could spell them out here. “the same models”: What does this mean?

Line 102: How many syllables for each word?

Line 107: How large was the dictionary?

Line 130: If you are calculating ERFs, please state that clearly.

Line 146: Why did you choose to use 2 different manipulations?

Lines 193-201: Please start with the simpler stimuli (e.g., envelope) to help the reader.

Line 204: Which phoneme alignment software did you use? That has to be specified. Was there manual alignment involved as well? Also, you talk about “phoneme features", but you are using the phonemes themselves as indicator variables. The papers you cite considered also “phonetic features” (e.g. fricative, plosive, voicing), so your referring to “features” at that particular point of the manuscript may sound confusing to some readers. Please clarify.

Lines 257 and 287: Please add an appropriate description of the filters (e.g., type and order).

Line 287: Does this mean that the low-pass filter was applied to data that had already been filtered? If that’s the case, this approach causes unnecessary additional noise due to the filtering artifacts. That could be easily avoided. Have the authors run any analysis to verify the robustness of these results to the change in filter type and order? The risk due to filtering artifacts must be considered (de Cheveigné and Nelken, Neuron, 2019).

Lines 307-308: You say “separately” twice. If that is not a typo, what do you mean by “separately for the spoken words”? By category? By participant? It’s not clear what you mean.

Line 312: Convolution -> Convolutional

Line 312: Isn’t your convolutional model a regression model (regularised linear regression - Pasley et al., 2012)? Please clarify that in both cases you use a regression model and that time-varying information was studies by including time-lags (e.g., lagged regression, multivariate temporal response function)

Line 316: You don’t say whether the phoneme information was standardised

Line 334: Did you use a published library or custom code? That should be stated.

You are using a lagged linear regression (convolution model) to reconstruct both continuous and categorical variables. This sounds particularly problematic for phoneme features, which are highly sparse. This sparsity issue is further complicated by the particular modelling approach, which seem to decode one variable at a time (one phoneme at a time). Have you considered using decoding algorithms that are more appropriate for categorical variables?

Given a stimulus with multiple dimensions, such as the phonemes, I understand that the “convolution model” reconstructs each feature at a time (e.g. phoneme 1 for all time points, phoneme 2 for all time points, and so on). How is the similarity score (Pearson’s correlation) calculated? By considering all values at once?

458: Why can’t the data be fully anonymised? 20) Line 508: Why not using forward models as it was done in the past [12]?

Line 544: “Fairly well”. I would avoid this kind of cloudy expressions.

Have you compared the result for the semantics descriptor with the ones when using a word onset (or uniqueness point), to be certain that the result depends on category processing rather than just word onset?

There is other relevant previous work that used approaches that are similar, in part, to yours. For example, Broderick et al, Curr Bio, 2018, who used word2vec as you do to assess semantic dissimilarity responses with EEG.

Figure 2.

x-axis: Please just report Time lag (ms) or Time latency (ms).

y-axis: The authors use a backward modelling approach, also called decoding model. For that reason, I find “reconstruction” or “decoding” more appropriate than “prediction”.

Figure 3: “Env. Sounds” stands for “environmental sounds”. Still, “Env.” Could be confused with “Envelope”. Please clarify.

Figure 5: I believe that the procedure to calculate the “spoken words” scores is clear. However, I am not sure I understand how the “Env. Sound” scores are calculated. What was reconstructed of the sounds in this case? The envelope or categorical information? And if the latter was true, how exactly did you do that? Categorical information was placed at the uniqueness point for spoken words. How was that done for environmental sounds?

Title: What does “dynamic” refer to exactly? To the fact that the inclusion of time-lags better explains the data for speech but not for env. Sounds? That should be clarified in the abstract and a bit more throughout the text

Audio files: Please present first the reconstruction and then the original sound. The current solution produces priming and, although it has been used in previous studies, it make the reconstructions sound better than they actually are. If you want to report them in this format, please indicate this issue somewhere in the text

## Author Response

Dear Editor and Reviewers,

We are grateful for your helpful suggestions to improve our work and the opportunity to revise our manuscript. The requested changes to the manuscript are visible in red font. Please find below our detailed responses to the comments.

Responses to major comments:

1. The interpretation that our brains use different processing modes for speech vs other sounds is very interesting, but I wonder whether this could be a trivial consequence of the used stimuli. It seems that environmental sounds can be identified or “comprehended” very early on (the semantic decoding analysis suggests after ∼ 50 to 100 ms), whereas words need to be analysed for a longer time to extract their meaning (the semantic decoding analysis suggests after 650 to 700 ms, and the uniqueness point is on average at 500 ms). Of course, the semantic decoding analysis cannot exactly reveal how much of the stimulus is needed to understand it, but it can serve as a proxy. This means that words need to be analysed ∼450-600 ms longer before a meaning can be extracted, thus the temporal tracking is necessary for a longer time because it adds new information. For other sounds no new information is inherent in the stimulus after the initial analysis of the sound. This would also mean, if environmental sounds were used that could only be identified after the same time as spoken words, the convolution model would be just as useful. In other words, there might well be two different modes, but they are not employed differently between words and other sounds, but they are employed depending on whether they are useful.

It is tricky to rule out this trivial explanation with the current stimuli, but one way I can think of is to repeat the analysis with a shorter time window (e.g. the first 100 ms of the words/sounds). With this shorter time window, the increased decoding performance from regression to convolution model should disappear (and if it is still there, and still not present for sounds, this would make a strong case for the author’s interpretation). Please report the results of this analysis and their interpretation in the reply and potentially in the manuscript.

Semantic decoding for spoken words was significant only late into the word, at 650-700 ms, whereas for environmental sounds semantic decoding remained significant from 150 ms on until the end of the analysis window (1000 ms). As you pointed out, this analysis cannot exactly reveal how much of the stimulus is needed to understand it. However, if it is taken as indication of identification time, it indicates that environmental sounds actually can also take a longer time to identify than spoken words, as semantic decoding can be performed based on the MEG signal at time points later than 650-700ms (the prediction was done separately for each 50ms time point and it was significant even at 950-1000 ms for the environmental sounds).

A more direct comparison of the spoken word and environmental sound identification time can be found in the one-back task, where participants had to indicate with finger lift, when two sounds had the same meaning. The results indicate that reaction times in sound identification were, in fact, longer for the environmental sounds (average RT for hits 1.7 s) than the spoken words (average RT for hits 1.5 s). This timing coincides with naming time for the environmental sounds in the pretest, which was also on average 1.7 s (1.1-2.9 s). These results indicate that it takes even a longer time to identify the environmental sounds than the spoken words. We have added these reaction time results in the manuscript (p. 22) and noted them in the discussion (p. 26).

Finally, as requested, we repeated the analysis with only the first 100 ms of the sound. For the convolution model, the spectrograms were cut to 0-100 ms length, and predicted from cortical responses with a lag window of 100-180 ms. This resulted in an average decoding accuracy of 69% for speech and 58% for environmental sounds, over the 16 participants (for the whole duration of the words and the same lag the decoding accuracies were 81% for speech and 61% for environmental sounds). Thus, although the decoding is much poorer with only the start of the word/sounds, as we expected, a significant difference remains between the spoken words and the environmental sounds (Z=3.16, p=0.002). This additional analysis shows that the enhanced decoding of words with the time-locked model is not explained by a longer analysis time that would be needed to extract word meanings, but the time-locked model applies from the start of the word. This result is now also reported in the manuscript (p. 21).

2. A second finding that the authors highlight is that “Time-locked encoding of spoken words reflects acoustic-to-phoneme mapping” (line 497). The underlying rationale is the finding by DiLiberto et al 2015. There have been some important developments regarding this finding in: Daube, C., Ince, R. A., & Gross, J. (2019). Simple acoustic features can explain phoneme-based predictions of cortical responses to speech. Current Biology, 29(12), 1924-1937. This study shows that phoneme-locking does not necessarily reflect a linguistic representation of phonemes, but is based entirely on acoustic features (i.e. the phoneme structure of speech is contained in the acoustic signal, and does not need to be “mapped” or “extracted”). The manuscript should include this development and discuss the implications in the discussion section.

Thank you for pointing out this new work for us. We do not wish to take a strong stand for either model, but we agree that also based on our results we do not need to assume a separate, phonological representation stage. As we stated in the abstract, the phoneme content was reflected in cortical evoked responses simultaneously with the spectrotemporal features, pointing to an instantaneous transformation of the unfolding acoustic features into linguistic representations during speech processing. The phoneme structure of speech is indeed contained in the acoustic signal and no separate prelexical stage of processing needs to be assumed. In any case, our results highlight the importance of temporal tracking for mapping speech acoustics to linguistic representations. We have now discussed this more and added Daube et al as reference (pp. 27-28).

3. The concepts of oscillations, entrainment and evoked responses are mixed up throughout the manuscript. Most notably this is seen on page 23, line 677: “phase-locked oscillations can be thought of as reflecting the superposition of transient evoked responses”. There is a current debate about the contribution of endogenous oscillations (which could be entrained by speech) versus only evoked responses (which occur to ANY sound). Evoked responses are not related to oscillations, and a time-locked mechanism does not “account for observations of oscillatory entrainment” (line 673). This distinction should be made clearer throughout.

We have modified this statement (p. 24) and the abstract, and tried to clarify the distinction between oscillatory entrainment and evoked responses. However, we do suggest that what may be regarded as oscillatory entrainment to speech, and time-locking of evoked responses to the speech signal may, at least partly, reflect the same underlying processes. Evoked responses have been shown to be related to phase-locked oscillatory activation, so that it can be modelled as a series of transient responses to changes in the stimulus (Aiken & Picton, 2008; see also a recent paper by Alexandrou et al, 2018). However, we do not deny the existence of endogenous oscillations that may be involved in speech processing.

4) The authors use a backward modelling analysis method, or decoding model, that allows to reconstruct a stimulus vector given the corresponding neural data. Specifically, the authors refer to this method as “regression” or “convolution” while, in fact, the method is just one. It’s a linear regression that, in some cases, uses a convolutional kernel that includes various time-latencies. However, the authors compare results for continuous and categorical vectors (e.g. speech spectrogram and phonemes), which is problematic as the model is tailored to continuous variables. Other approaches (e.g. logistic regression) would be better suited for this application. In fact, the authors measured much lower “prediction” scores for categorical (phonemes and semantics) than continuous variables. However, they don’t seem to consider the method itself as a potential cause for this result. This is an issue, as part of the discussion is centred on this result. Similarly, the manuscript could be clearer on how exactly the “prediction” scores were calculated for categorical variables.

You are correct, the convolution model is a regularized regression with lagged windows. For simplicity, we refer to this model as the convolution model. We have now indicated this in the Methods (p. 12). As to how the decoding scores were calculated; first, reconstruction of each feature was done separately (e.g. each frequency band of the spectrogram, each phoneme or each semantic feature). The similarity scores (correlations between original and reconstructed features) were calculated by considering all values at once (all phonemes, all frequency bands etc.). This is described in the Methods (pp. 13 & 15).

Indeed, our model was not optimized for phoneme decoding (categorical variable), but as a post-hoc analysis we wanted to investigate whether the result of time-locking to the speech spectrogram could reflect phoneme decoding and if the latency of this decoding would be the same or if it would differ in timing. Therefore, we used the exact same model. We were not very interested in comparing the results for continuous and categorical vectors (i.e. for which feature set, phonemes, semantics or acoustics is the decoding better), or to find physiologically interpretable response patterns for each phoneme. We acknowledge that other approaches might be better suited for finding optimal reconstruction performance for categorical phonemes (see response to point 6 below). This is now indicated in the text (pp. 14, 18 & 27-28).

The decoded semantic features are not categorical and not particularly sparse; we use two sources of information to form the semantic features, question norms and corpus statistics. The former is an average of responses to 99 questions across 59 participants - each response is on a scale from one to five and the averaging across 59 participants makes the features continuous. The latter (corpus statistics derived norms), are based on word-2-vec representation of each word with dimensionality 300 which is again continuous. The final semantic representation is the concatenation of 99 question norms with the 300 corpus derived norms (as combination of the two norms proved to better perform the decoding compared to either of the two norms considered separately).

5) Why was the decoding analysis done in sensor space instead of source space?

The analysis was done both in sensor space and in source space. Because of high dimensionality, to reduce computational time, the source space analysis was restricted to the models and time windows that performed best for each stimulus type in sensor space (MPS-regression-based acoustic decoding for environmental sounds, spectrogram-convolution-based acoustic decoding for spoken words, and regression-based semantic decoding for both). Cortical sources contributing to decoding are illustrated in Fig. 2b, 3c and 5b (see also Fig. 5-1).

6) The modelling approach has at least other two issues that could be easily solved and should be discussed. First, the regression (“convolution model”) constitutes a backward model fit that decodes a stimulus vector given the corresponding MEG signal. However, the mathematical formulation indicates that the reconstruction is performed on each feature independently (e.g., phoneme A, phoneme B; multivariate-to-univariate), which is particularly problematic for sparse categorical variable. Approaches such as Canonical Correlation Analysis (for example, see the work from Alain de Cheveigné’s lab and Lucas Parra’s lab) would solve this issue as they are multivariate-to-multivariate mapping methods. This issue should be discussed as it can also explain why the results for the envelope are much better than the ones for the phoneme descriptor. The second issue regards the study of the temporal latencies of the effects. While the approach adopted by the authors is valid, it is certainly suboptimal. A more reasonable approach would be, instead, to reverse the regression approach to fit forward models, which are instead directly physiologically interpretable and take into account all stimulus features and all time-latencies at the same time. Note that backward models can also be mathematically transformed into the corresponding forward models (Haufe et al., NeuroImage, 2014).

Indeed, our model was not optimized for phoneme decoding (categorical variable), which can explain why the results for the spectrogram and envelope are better than the ones for the phoneme descriptor. This is now indicated in the text (pp. 14, 18 & 27-28).

The backward model adopted in this study is motivated by allowing that it is sensible to assume neural response at different areas “x” within auditory cortex jointly work together to predict an individual stimulus feature. A forward model would be much simpler however it would assume that neural responses at different brain locations “r(x)” are independent which seems a more strict assumption compared to the backward model.

Other methods such as CCA would indeed be more suitable for categorical variables, and forward models especially for finding spatially and temporally unique, physiologically interpretable neural response patterns for each phoneme. However, our goal was simply to see, whether the spectrogram convolution decoding could reflect categorical phoneme features, and whether the timing (optimal lag) is similar for acoustic and phoneme features. We were primarily interesting in a unique (potentially interpretable) encoding mechanism for spoken words compared to environmental sounds. The basic and mathematically simplistic encoding model is the linear regression, where the degrees of freedom are high i.e., the model is flexible to uncover possible multivariate patterns among the neural response features that together decode a univariate acoustic feature. The convolution model is more constrained with lesser degrees of freedom, and particularly the neural responses are constrained (model parameters; g_f (τ,x) are not time-dependent in the neural response but rather shared among the responses r(t,τ,x) having the same lag τ. Effectively, this allows us to study if the specific assumption of temporal tracking is more suitable to decode environmental sounds versus spoken words. It would be interesting to study if Canonical correlation analysis, especially a suitable constrained version, would offer more accurate reconstruction of the spectrogram, amplitude envelope or phoneme sequence.

7) Based on my previous comments, several discussion points should be toned-down. The current analysis has issues that may undermine some of those interpretations.

We have modified the discussion accordingly, especially regarding the phoneme decoding (p. 27-28).

Responses to minor comments:

The study had only 16 participants (and no a priori estimated sample size), which is becoming less and less acceptable in psychological and neuroimaging studies (e.g. Simmons JP, Nelson LD, Simonsohn U (2011) False-positive psychology: Undisclosed flexibility in data collection and analysis allows presenting anything as significant. Psychological science 22: 1359-1366. https://doi.org/10.1177/0956797611417632). While it would be unfair to request additional data, a sample size of 16 is not something that should be advocated, particularly in a wide-reaching journal like eNeuro. Please add an explicit justification for this low number of participants and demonstrate awareness that this is not good scientific practice.

Our target sample size was 20, but in the end only 3 pilots and 16 participants were measured, because the results were already quite clear. We do not consider 16 participants to be a very small sample, especially given the robustness of the effects of interest. The main result, i.e. the benefit of the convolution model compared to the non-time-sensitive models for speech, was apparent in all participants, and had a very large effects size (r=0.88). Below you can see the individual decoding accuracies for these two models for speech. In other tests the effect sizes ranged from medium to large.

MPS Regression Spectrogram Convolution Improvement in decoding

s01 53,91 82,24 28,33

s02 66,28 88,27 21,99

s03 64,80 76,22 11,42

s04 72,83 81,92 9,09

s05 67,44 86,05 18,60

s06 72,41 81,92 9,51

s07 72,30 86,79 14,48

s08 64,38 94,50 30,13

s09 69,34 83,09 13,74

s10 69,13 89,01 19,87

s11 55,39 70,19 14,80

s12 70,51 86,58 16,07

s13 67,76 81,08 13,32

s14 59,83 90,06 30,23

s15 58,25 83,09 24,84

s16 59,62 73,47 13,85

AVERAGE 65 83 18

SEM 1,54 1,56 1,77

On page 21, line 613, there appears to be a randomly chosen p-value (“Bonferroni corrected significance limit 0.0125”). Please justify this based on similar previous analyses.

This statement meant to say that p-values less than 0.05/4=0.0125 are acceptable. It has been clarified.

Which programme/software was used for the decoding analyses?

The decoding analysis was implemented in Matlab with our in-house scripts. The code is available in github (https://aaltoimaginglanguage.github.io/speechness).

The conclusion starts with “The present discovery of a dynamic, time-locked mechanism for speech processing”. The faithful, “time-locked” tracking of speech and other sounds is well-established and I wouldn’t go as far as calling it a discovery. The thorough comparison between speech and other sounds is the novel aspect of this study, in my opinion.

We agree, and have modified this statement accordingly.

The final sentence of the conclusion (page 28, line 818) states: “the present findings offer a crucial link between investigations of cortical processing of continuous speech and isolated linguistic stimuli”. How does this study offer a link between both when it only addresses isolated words and sounds?

This statement refers to the possible link between the widely reported oscillatory entrainment to continuous speech, and the time-locking of evoked responses to speech, as observed in this study. This statement has been clarified.

Line 75: “His” -> “This”?

Corrected.

Line 81: Those studies showed that neural signals reflect phoneme responses, true. However, saying that phonemes have been successfully decoded sounds like an overstatement if not clarified. “Successfully decoded” could sound as if can already build a brain-to-speech device, while that’s not true, not yet. Please clarify.

We agree. We have clarified this statement.

Lines 82-84: This is a very important point that clarifies the novelty of this work. I feel that this sentence can be improved to make your point more strongly. For example, “all of these levels of analysis”: You could spell them out here. “the same models”: What does this mean?

We agree that this is the biggest novelty in our study. We have tried to clarify this statement.

Line 102: How many syllables for each word?

If this question refers to the uniqueness points, they varied from the first to the fourth syllable.

Line 107: How large was the dictionary?

The dictionary was a 1.5 billion-token Finnish Internet-derived text corpus.

Line 130: If you are calculating ERFs, please state that clearly.

This is now stated more clearly (p. 5).

Line 146: Why did you choose to use 2 different manipulations?

We processed the sounds as long as we considered them unidentifiable (reversed them, and in some cases also, after this, scrambled them). All the sounds were tested for naming, and the once that were not named consistently were chosen as meaningless sounds.

Lines 193-201: Please start with the simpler stimuli (e.g., envelope) to help the reader.

The order has been changed as requested (p. 7).

Line 204: Which phoneme alignment software did you use? That has to be specified. Was there manual alignment involved as well? Also, you talk about “phoneme features", but you are using the phonemes themselves as indicator variables. The papers you cite considered also “phonetic features” (e.g. fricative, plosive, voicing), so your referring to “features” at that particular point of the manuscript may sound confusing to some readers. Please clarify.

The phoneme categories were aligned manually with Praat software. We tested also automatic alignment methods, but they did not function well enough for our stimuli. By “features” we refer to each of the feature sets that we are decoding. We agree that this terminology can be confusing, so we have tried to clarify this in this section (p. 7).

Lines 257 and 287: Please add an appropriate description of the filters (e.g., type and order).

Line 287: Does this mean that the low-pass filter was applied to data that had already been filtered? If that’s the case, this approach causes unnecessary additional noise due to the filtering artifacts. That could be easily avoided. Have the authors run any analysis to verify the robustness of these results to the change in filter type and order? The risk due to filtering artifacts must be considered (de Cheveigné and Nelken, Neuron, 2019).

Online filtering of the raw data during measurement (here between 0.03 and 330 Hz) for anti-aliasing and avoiding low-frequency artifacts in the data is standard procedure in MEG studies. In Elekta Neuromag Acquisition, there is an analog high-pass filter and a 6th order Butterworth IIR low-pass filter (DSP-based).

The filtering of the averaged responses at 40 Hz was done only for visualization of the data, and this is now indicated in the text. We acknowledge that this might potentially create artifacts (e.g. change the timing of event in evoked responses), but the filters were chosen so as to avoid these. We have now described the filters in more detail (pp. 9 & 10).

Lines 307-308: You say “separately” twice. If that is not a typo, what do you mean by “separately for the spoken words”? By category? By participant? It’s not clear what you mean.

Each decoding model was trained and tested separately within each individual participant (line 305). Also, training and testing were done within spoken words or environmental sounds, not mixed together. We have clarified this statement (p. 11).

Line 312: Convolution -> Convolutional

Corrected

Line 312: Isn’t your convolutional model a regression model (regularised linear regression - Pasley et al., 2012)? Please clarify that in both cases you use a regression model and that time-varying information was studies by including time-lags (e.g., lagged regression, multivariate temporal response function).

You are correct, the convolution model is a regularized regression with a lagged windows. For simplicity, we refer to this model as the convolution model. We have now indicated this in the Methods (p. 12). The lagged regression model (as in Pasley et al) usually only indicates a single parameter tau which is lagged with respect to the current time specified in the dependent variable. We present our model from the perspective of convolution model literature, where the idea of convolving an unknown response function with neural response is used to reconstruct the stimuli, and in this respect the convolution window (t-τ_2) to (t-τ_1) becomes a more interesting way to interpret the potential uniqueness of spoken words vs environmental sounds.

Line 316: You don’t say whether the phoneme information was standardised

The phoneme sequence was a categorical variable (multiple time series with values 0/1), so it was not standardized.

Line 334: Did you use a published library or custom code? That should be stated.

Sound analysis and reconstruction was conducted with the freely available NSL toolbox (http://www.isr.umd.edu/Labs/NSL/Downloads.html). The PCA analysis for removing blink artefacts from MEG signals was done with custom-made scripts using the Neuromag (MEGIN) software. Other preprocessing and source modeling were done using standard MEG analysis software and scripts (Neuromag, MNE Suite). MR image surface reconstruction was done with Freesurfer. The machine-learning methods have been performed with our custom-made Matlab and shell scripts (now stated on p. 16), which is available in github (https://aaltoimaginglanguage.github.io/speechness).

You are using a lagged linear regression (convolution model) to reconstruct both continuous and categorical variables. This sounds particularly problematic for phoneme features, which are highly sparse. This sparsity issue is further complicated by the particular modelling approach, which seem to decode one variable at a time (one phoneme at a time). Have you considered using decoding algorithms that are more appropriate for categorical variables?

The convolution model was not optimized for phoneme decoding (categorical variable), but as a post-hoc analysis, we wanted to investigate, whether the time-locking to the speech signal could reflect phoneme decoding. We acknowledge that other approaches might be better suited for this particular application (see response to major comments 4 and 6 above). This is now mentioned in the text (pp. 14, 18 & 27-28).

Given a stimulus with multiple dimensions, such as the phonemes, I understand that the “convolution model” reconstructs each feature at a time (e.g. phoneme 1 for all time points, phoneme 2 for all time points, and so on). How is the similarity score (Pearson’s correlation) calculated? By considering all values at once?

You are correct; reconstruction was done for each feature separately, and the similarity scores (correlations between original and reconstructed values) were calculated by considering all values (all phonemes, all frequency bands etc.) at once.

458: Why can’t the data be fully anonymised?

The collected MEG data include direct identifiers and indirect identifiers of the participants. The data has been stripped of direct identifiers such as personal information. However, research from our laboratory shows that any MEG data contains indirect identifiers, and participants can be identified solely based on the rhythmic structure within the data (Leppäaho et al, Human Brain Mapping 2019: 40, 139402). Thus, at best, we think that the data may be pseudonymized. More importantly, the Finnish law and the requirements defined by Aalto University Research Ethics Committee currently limit the use of brain imaging data only for purposes for which a written consent was given by the participants (data collected before the current EU-wide GDPR). Our data officers are looking into means to make data publicly available, but as the use of the data cannot be restricted if it is placed in a public repository, currently our MEG data is only available from the authors upon reasonable request for research purposes.

Line 508: Why not using forward models as it was done in the past [12]?

See response to major comment 6.

Line 544: “Fairly well”. I would avoid this kind of cloudy expressions.

Corrected.

Have you compared the result for the semantics descriptor with the ones when using a word onset (or uniqueness point), to be certain that the result depends on category processing rather than just word onset?

If you mean that our semantic features would not be represented in the data, but instead what we are decoding are differences in word onsets (acoustic features), we do not think this can be the case. This is because the same set of semantic features was successfully decoded for both the environmental sounds and spoken words, with different acoustics, sound onsets and identifications times.

However, there is one way in which the acoustic features may contribute to the semantic decoding: environmental sounds typically have more variation across categories than within categories, as sounds with a similar source are more likely to share acoustic features such as frequency content and degree of harmonics or periodicity (e.g. animal vocalizations vs. tool sounds). This could be reflected in the MEG responses and, in theory, improve the semantic decoding of environmental sounds on category level. To investigate this possibility, we compared the dissimilarity matrices of the semantic features and acoustic features (se figure below) for both stimulus groups with a Mantel test. Some overlap was found in the category structures of acoustic and semantic features for environmental sounds (p = 0.004) but not for spoken words (p = 0.80). Thus, for environmental sounds, the high semantic decoding may partly originate from different acoustic features between the different sound categories. This is possibly reflected in the category-level decoding of semantic features that was significantly better than item-level decoding particularly for environmental sounds (Z = 3.4, p = 0.00015). However, the better performance for category-level than item-level decoding was also observed to some extent for spoken words (Z = 2.2, p = 0.025), so it was not entirely due to acoustic within-category similarity and between-category differences (in environmental sounds); it seems to be due to salient cortical representations of the semantic categories.

There is other relevant previous work that used approaches that are similar, in part, to yours. For example, Broderick et al, Curr Bio, 2018, who used word2vec as you do to assess semantic dissimilarity responses with EEG.

Thank you for pointing out this interesting work. We have now referred to it in our manuscript (pp. 3 & 26).

Figure 2.

x-axis: Please just report Time lag (ms) or Time latency (ms).

y-axis: The authors use a backward modelling approach, also called decoding model. For that reason, I find “reconstruction” or “decoding” more appropriate than “prediction”.

We agree. We have corrected this in all the figures and many places in the text.

Figure 3: “Env. Sounds” stands for “environmental sounds”. Still, “Env.” Could be confused with “Envelope”. Please clarify.

We have corrected this in all the figures.

Figure 5: I believe that the procedure to calculate the “spoken words” scores is clear. However, I am not sure I understand how the “Env. Sound” scores are calculated. What was reconstructed of the sounds in this case? The envelope or categorical information? And if the latter was true, how exactly did you do that? Categorical information was placed at the uniqueness point for spoken words. How was that done for environmental sounds?

The uniqueness points for words were not used in the semantic decoding analysis, they just happen to coincide with the significant semantic decoding observed for spoken words. Instead, semantic decoding was done for all 50 ms time windows of the MEG signal, and Fig. 5 depicts the time windows where decoding was significant. This is now clarified in the figure legend.

The semantic features and their decoding were the same for both environmental sounds and spoken words. In semantic feature decoding, the reconstructed features were the answers to questions (with values on scale 1-5) and corpus vectors (with continuous values), not categorical information about the semantic category. Decoding was done on category level, i.e. by always telling apart two sounds from different categories. We apologize if this was unclear, it has been clarified in the Results (pp. 22-23).

Title: What does “dynamic” refer to exactly? To the fact that the inclusion of time-lags better explains the data for speech but not for env. Sounds? That should be clarified in the abstract and a bit more throughout the text

Here “dynamic” refers to encoding/decoding of the spoken words, that is moving in time, i.e. tightly following the stimulus acoustics. The cortical responses are dynamically changing in response to the stimulus, with a short lag.

Audio files: Please present first the reconstruction and then the original sound. The current solution produces priming and, although it has been used in previous studies, it makes the reconstructions sound better than they actually are. If you want to report them in this format, please indicate this issue somewhere in the text.

This issue has now been indicated in the legend for the Audio files.
