# Supplementary material for: Dynamic Time-Locking Mechanism in the Cortical Representation of Spoken Words
Source: eNeuro. 2020 Jul 22;7(4):ENEURO.0475-19.2020. doi: 10.1523/ENEURO.0475-19.2020 (PMC7470935; doi:10.1523/ENEURO.0475-19.2020)
Supplement: Extended Data Table 1-1 — Table containing a complete list of stimulus items. Download Table 1-1, DOC file. [file enu-eN-NWR-0475-19-s07.doc]

**Extended Data Table 1-1.** List of stimuli: All items had one spoken word and one environmental sound exemplar.

| **CATEGORY**  ANIMAL  HUMAN SOUND  MUSICAL INSTRUMENT  TOOL  VEHICLE  OTHER | **ITEM**  Horse  Chicken  Cat  Dog  Sheep  Cow  Bird  Pig  Sneeze  Yawn  Crying  Laughing  Vomiting  Burping  Whistling  Coughing  Accordion  Flute  Guitar  Piano  Drums  Trumpet  Organ  Violin  Chainsaw  Drill  Saw  Match  Hammer  Knife  Ambulance  Car  Helicopter  Train  Ship  Airplane  Door  Doorbell  Telephone  Thunder  Water  Zipper  Billiard  Camera | **SPOKEN WORD (Finnish)**  hevonen  kana  kissa  koira  lammas  lehmä  lintu  sika  aivastus  haukotus  itku  nauru  oksennus  röyhtäys  vihellys  yskä  haitari  huilu  kitara  piano  rummut  trumpetti  urut  viulu  moottorisaha  porakone  saha  tulitikku  vasara  veitsi  ambulanssi  auto  helikopteri  juna  laiva  lentokone  ovi  ovikello  puhelin  ukkonen  vesi  vetoketju  biljardi  kamera | **ENVIRONMENTAL SOUND**  [horse neigh]  [chicken bwaak-bwak-bwak]  [cat meow]  [dog bark-bark]  [sheep baa]  [cow moo]  [little songbird chirping]  [pig oink-oink]  [female sneezing achoo]  [male yawning]  [female crying]  [female laughing]  [male (mimicking) vomiting]  [male burping]  [male whistling]  [male coughing]  [accordion chord]  [three descending flute notes]  [two chords on guitar]  [two chords on piano]  [one cycle of drum beat]  [ascending trumpet tune with six notes]  [organ chord]  [violin note with bow-change]  [chainsaw running]  [drill running on air]  [three draws of a hand-held saw]  [lighting a match]  [two strikes of a hammer]  [sound of sharpening a knife]  [ambulance siren]  [car starting]  [helicopter circulating]  [train hitting the tracks]  [ship foghorn]  [airplane landing]  [door closing heavily]  [doorbell ding-dong]  [a traditional telephone ring]  [thunder rolling afar]  [stream of water]  [zipper closing]  [billiard balls hitting each other]  [traditional camera shutter sound] |
| --- | --- | --- | --- |
